# Characterization of rumen, fecal, and milk microbiota in lactating dairy cows

**DOI:** 10.3389/fmicb.2022.984119

**Published:** 2022-09-26

**Authors:** Jenna R. Williamson, Todd R. Callaway, Jeferson M. Lourenco, Valerie E. Ryman

**Affiliations:** Department of Animal and Dairy Science, University of Georgia, Athens, GA, United States

**Keywords:** microbiome, rumen, fecal, milk, dairy cattle, somatic cell count, milk yield, intramammary

## Abstract

Targeting the gastrointestinal microbiome for improvement of feed efficiency and reduction of production costs is a potential promising strategy. However little progress has been made in manipulation of the gut microbiomes in dairy cattle to improve milk yield and milk quality. Even less understood is the milk microbiome. Understanding the milk microbiome may provide insight into how the microbiota correlate with milk yield and milk quality. The objective of this study was to characterize similarities between rumen, fecal, and milk microbiota simultaneously, and to investigate associations between microbiota, milk somatic cell count (SCC), and milk yield. A total of 51 mid-lactation, multiparous Holstein dairy cattle were chosen for sampling of ruminal, fecal, and milk contents that were processed for microbial DNA extraction and sequencing. Cows were categorized based on low, medium, and high SCC; as well as low, medium, and high milk yield. Beta diversity indicated that ruminal, fecal, and milk populations were distinct (*p* < 0.001). Additionally, the Shannon index demonstrated that ruminal microbial populations were more diverse (*p* < 0.05) than were fecal and milk populations, and milk microbiota was the least diverse of all sample types (*p* < 0.001). While diversity indices were not linked (*p* > 0.1) with milk yield, milk microbial populations from cows with low SCC demonstrated a more evenly distributed microbiome in comparison to cows with high SCC values (*p* = 0.053). These data demonstrate the complexity of host microbiomes both in the gut and mammary gland. Further, we conclude that there is a significant relationship between mammary health (i.e., SCC) and the milk microbiome. Whether this microbiome could be utilized in efforts to protect the mammary gland remains unclear, but should be explored in future studies.

## Introduction

Studying the microbiome allows us to understand which microbes live in or on animals, and the two most studied ecosystems in cattle are the ruminal and fecal microbiomes (Dowd et al., 2008). Cattle rely on their rumen for fermentation of feedstuffs into nutrients and energy (Matthews et al., 2019), which is the largest compartment of the gastrointestinal tract (GIT) and is home to an extensive microbial population that degrades feedstuffs via fermentation. Ruminal fermentation is performed by bacteria, archaea, protozoa, and fungi, which can be manipulated through diet supplementation to reduce production costs, promote feed efficiency, and improve animal health (Lourenco et al., 2020). There is a clear association between rumen and fecal microbiota and feed efficiency in cattle (Shabat et al., 2016), which translates into reduced feed costs as the animal maintains a more adequate body weight but consumes less feed (Schären et al., 2018; Lourenco et al., 2020; Welch et al., 2020). Additionally, microbiome populations are also correlated with and/or contribute to health and disease, such as metabolic disorders, cow fertility, GIT dysfunctions, and inflammation (Matthews et al., 2019; O’Hara et al., 2020; Xu et al., 2021).

Despite the volume of information on the GIT microbial population, little remains known about how milk yield and milk quality can be impacted by manipulation of the gut microbiota in dairy cattle. Aside from limited assessments of feed efficiency and milk production variables such as lactation stage and milk composition (Schären et al., 2018; Xue et al., 2018, 2022; Buitenhuis et al., 2019; Hagey et al., 2019), much of the dairy cattle microbiome research has focused on mitigating GIT inflammation or reducing greenhouse gas emissions (Sanz-Fernandez et al., 2020; John et al., 2022). Few studies have evaluated the associations between the rumen microbiome and milk yield or quality (Xue et al., 2018; Buitenhuis et al., 2019; Hagey et al., 2019). Even fewer studies have investigated the fecal microbiome of dairy cattle; however, reports suggested potential differences in the rumen and fecal populations that may explain differences between low-producing vs. high-producing dairy cattle (Mu et al., 2019). Yet, there is still another microbial environment to be explored in dairy cattle that may be more important to milk production than the GIT populations.

For decades, milk was assumed to be sterile unless contaminated or infected, such as during intramammary infections (IMI) caused by mastitis pathogens. However, Next-Generation Sequencing techniques have revealed that even when the mammary gland is free from culturable pathogens, milk has its own resident microbial population, with the vast majority of these organisms not associated with mastitis (Addis et al., 2016; Derakhshani et al., 2018). Understanding the normal composition of milk microbiome may reveal how these microbial communities are related to both milk yield (low vs high) and milk quality. Milk quality can be measured through a variety of assessments; however, the most common parameter utilized is somatic cell counts (SCC; Alhussien and Dang, 2018). The SCC of milk is directly related to milk quality; when SCC values are high (typically associated with IMI), the composition of milk is negatively impacted (Ballou et al., 1995). A healthy mammary gland should have a SCC < 200,000 cells/ml, while the presence of IMI typically causes SCC values to rise above 500,000 cells/ml, potentially resulting in the need for antibiotic treatment to recover mammary health. Identification of a milk microbiome that is associated with desirable production values such as high milk yield and low SCC, along with conferring protection against infectious mammary pathogens would be pivotal in minimizing antibiotic usage on dairy farms. Distinct populations of the milk microbiome are expected, as milk is an aerobic environment with different nutrients available for microbial growth and little competition in comparison to the anaerobic GIT environment (Hungate, 1966). In addition, to further understand how the milk microbiome may be established. There are several possibilities of biological pathways/methods of transfer in forming the milk microbiota, including: (a) an entero-mammary pathway where bacteria may be translocated from the GIT to the mammary gland by immune cells (Rodríguez, 2014; Ma et al., 2018; Hu et al., 2022), (b) the milk or mammary gland is inoculated with feces or other environmental sources (Doyle et al., 2016), or (c) the oral cavity of the calf contributes to milk microbiota formation of the lactating dam (Williams et al., 2019; Oikonomou et al., 2020). Therefore, the objective of the present study was to characterize rumen, fecal, and milk microbiota simultaneously, and identify relationships between microbial communities, milk SCC, and milk yield.

## Materials and methods

All procedures involving live animals were verified and approved by the University of Georgia’s Office of Animal Care and Use (AUP #A2021 07-029-Y1-A0). The dairy cattle used in this study were located at the University of Georgia Teaching Dairy in Winterville, GA (33°54′32.9″N 83°14′50.9″W).

### Animal selection and collection

A total of 51 Holstein dairy cattle were selected from the UGA Teaching Dairy. Animals chosen were clinically healthy, in their 2nd or greater lactation (multiparous), and within 30–305 days in milk. Cows outside these requirements may be more metabolically stressed and experiencing greater immunological challenges that could influence the microbiome (De Haas et al., 2002; Alhussien and Dang, 2018), and therefore were not included in the study. Cows were housed in a free stall barn using sand bedding that is rebedded every 1–2 weeks and milked twice daily. Ration composition is available in Supplementary Tables 1, 2.

Rumen fluid (*n* = 51) was collected from all cows 2–4 h post feeding following procedures described by Lourenco et al. (2019). The first 200 ml of ruminal fluid obtained was discarded to avoid saliva contamination. Following, approximately 300 ml of ruminal fluid was collected from each animal by esophageal tubing using an electric vacuum pump and weighted perforated metal probe, which was sanitized between animals to avoid contamination. Immediately after, a subsample of 40 ml was transferred to a sterile 50 ml conical vial and placed immediately on ice. Samples were transported to a −20°C freezer for long-term storage until deoxyribonucleic acid (DNA) extraction could be performed ~3 months later.

Fecal samples (*n* = 51) were collected by digital palpation from all cows as described previously by Lourenco et al. (2020). Cows were rectally palpated to collect fecal material that was then transferred to a sterile 50 ml conical vial and placed immediately on ice. Samples were transported to a −20°C freezer for long-term storage as described above.

Milk from mammary quarters of cows (*n* = 47) were aseptically collected into sterile 50 ml conical vials. Each teat of the mammary gland was wiped with a gloved hand to remove loose dirt and debris and 3–5 streams of milk were stripped from each quarter. Any abnormalities in the milk or teat/mammary gland were noted, if any present. Each teat was then dipped in a germicidal dip containing 1% iodine. After 30 s, the germicidal dip was wiped completely from the teats and teat ends, and teat ends were then scrubbed vigorously with a cotton ball soaked in 70% isopropyl alcohol. Approximately 12 ml of milk from each quarter was expressed to create a composite sample. Samples were vortexed and immediately placed on ice for transport to a −20°C freezer for long-term storage as described above. In addition to the milk samples collected in 50 ml conical vials, another composite sample was collected into a sterile tube and immediately placed on ice. These tubes were transported to the UGA Mastitis Lab the same day as collection for SCC enumeration. The additional sample was taken to avoid contamination of the prior sample for DNA extraction, as contamination of samples with low biomass can dominate the majority of sequences observed in a microbiome analysis (Salter et al., 2014; Kim et al., 2017). The SCC of the composite sample was determined using a DeLaval Direct Cell Counter (DeLaval; Tumba, Sweden) and SCC was recorded for all samples. Cows were categorized based on milk SCC into three SCC groups for comparison with milk microbiota: low = SCC ≤ 200,000 cells/ml, medium = 201,000 cells/ml < SCC < 800,000 cells/ml, high = SCC ≥ 800,000 cells/ml. Additionally, the average daily milk yield was collected for each cow from the day prior to sample collection. Cows were categorized based on daily milk yield (lbs/day) into one of three production levels: low = ≤65 lbs, medium = 65 lbs < x < 90 lbs, high = ≥90 lbs.

### DNA extraction of rumen, fecal, and milk contents

Microbial DNA from the ruminal, fecal, and milk samples was extracted following the procedures previously described by Welch et al. (2020) with modifications. Briefly, 350 μl of rumen sample, 0.35 g of fecal sample, or 1,000 μl of milk sample were placed in 2-ml Lysing Matrix E tubes (MP Biomedicals LLC, Irvine, CA, United States), which are homogenized using a QIAGEN vortex adapter (QIAGEN, Venlo, Netherlands) to disrupt the cells. Enzymatic inhibition was achieved by using InhibitEX Buffer (QIAGEN, Venlo, Netherlands), and DNA elution and purification were carried out using a spin column and a series of specialized buffers according to manufacturer’s specifications (QIAamp Fast DNA Stool Mini Kit; QIAGEN, Venlo, Netherlands). Calculation of DNA concentration and purity in the resulting eluate was performed spectrophotometrically using the Synergy LX Multi-Mode Microplate Reader in conjunction with the Take3 Micro-Volume Plate (BioTek Instruments Inc; Winooski, VT, United States). Samples with a minimum volume of 90 μl and 10 ng/μl of DNA were stored at −80°C until the following day. Samples that failed to meet these requirements were rejected and subjected to a new DNA extraction cycle.

### DNA extraction of milk contents

Due to the lack of identifiable DNA in some milk samples using the previous QIAGEN kit, another DNA kit was used that was more appropriate for samples of low biomass. This procedure uses 1,000 μl of sample placed in 2-mL PowerBead Tubes (QIAGEN, Venlo, Netherlands), which are heated in a water bath and then homogenized using a QIAGEN vortex adapter (QIAGEN, Venlo, Netherlands) to disrupt the cells. The samples were then centrifuged at 10,000 × *g* for 1 min to remove debris and PowerBeads from the supernatant before Solution IRS (QIAGEN, Venlo, Netherlands) was used to remove all inhibitors from the sample. Using an MB Spin Column and a series of specialized buffers according to manufacturer’s specifications (QIAamp BiOstic Bacteremia DNA Kit; QIAGEN, Venlo, Netherlands), genomic DNA was eluted and purified. The concentration and purity of DNA in the resulting eluate was determined spectrophotometrically, using the Synergy LX Multi-Mode Microplate Reader in conjunction with the Take3 Micro-Volume Plate (BioTek Instruments Inc; Winooski, VT, United States). Samples with a minimum volume of 90 μl and 10 ng/μl of DNA were stored at −80°C until the following day, per recommendation by the manufacturer (QIAGEN, Venlo, Netherlands) as the final solution does not contain EDTA that prevents the degradation of DNA. Any samples that failed to meet these requirements were rejected and the DNA extraction cycle was repeated.

### DNA data sequencing analysis

Following DNA extraction, samples were shipped overnight on dry ice to LC Sciences (Houston, TX, United States; https://lcsciences.com/services/dna-sequencing/microbial-sequencing/) for library preparation and 16S ribosomal ribonucleic acid (rRNA) gene sequencing. The library preparation step included polymerase chain reaction (PCR) replications using the forward: S-D-Bact-0341-b-S-17 (5′-CCTACGGGNGGCWGCAG-3′) and reverse: S-D-Bact-0785-a-A-21 (5′-GACTACHVGGGTATCTAATCC-3′) primer pairs (Klindworth et al., 2013), followed by a PCR clean-up using AMPure XP beads (Beckman Coulter Life Sciences, Indianapolis, IN, United States). A second PCR step was then carried out to attach Illumina’s indices and sequencing adapters (Nextera XT Index Kit; Illumina Inc., San Diego, CA, United States), followed by another PCR clean-up step using AMPure XP beads. Following this final library clean up, the library was quantified using qPCR, and the nucleotides were sequenced using an Illumina NovaSeq instrument and a NovaSeq v2 reagent kit (Illumina Inc., San Diego, CA, United States). A well-characterized bacteriophage PhiX genome (PhiX Control v3 Library; Illumina Inc., San Diego, CA, United States) was used as a control for the sequencing runs.

Sequencing data were first demultiplexed before being converted into FASTQ files, and the paired-end sequences imported into QIIME 2 (Bolyen et al., 2019). The non-biological nucleotides were then removed, and sequences were denoised, dereplicated, and chimera-filtered using DADA2 (Callahan et al., 2016). Taxonomies were assigned to the sequences by using a pre-trained Naive Bayes classifier trained on the SILVA 138 SSU database (Quast et al., 2013), and reads were classified by taxon using the fitted classifier (Pedregosa et al., 2011). For further analysis, the sequencing depth was set at 27,230 sequences per sample. The following alpha-diversity indices were computed for each sample: Shannon Diversity Index, Faith’s Phylogenetic Diversity Index, Pileou’s Evenness Index, and number of amplicon sequence variants (ASVs). For beta diversity, we present results only from the unweighted UniFrac distance matrix. Additionally, relative bacterial abundances were quantified at the phylum and genus levels.

### Statistical analysis

Statistical analyses were performed using the software Minitab^®^ v21 (Minitab LLC, State College, PA) with all models corrected for the DNA kit used. The alpha diversity indices in rumen, fecal, and milk samples were analyzed using the non-parametric Kruskal-Wallis test with milk SCC and milk yield as factors. Analyses of relative bacterial abundances at the phylum and genus levels in rumen, fecal, and milk samples were carried out by Kruskal-Wallis using milk SCC and milk yield as factors. The differences in beta-diversity were assessed using two-sample *t*-tests. All *p*-values were corrected by Bonferroni’s method for multiple comparisons. For all the statistical tests used, results were considered significant at *p* ≤ 0.05 and were treated as trends when 0.05 < *p* < 0.10.

## Results and discussion

### Microbial diversity

**Figure 1:**
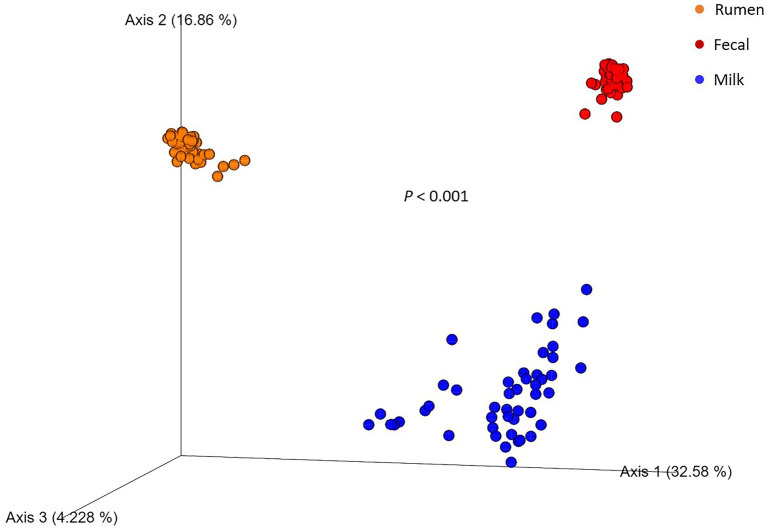
Principal coordinate analysis plot of beta diversity of rumen (*n* = 51), fecal (*n* = 51), and milk (*n* = 47) microbial populations of Holstein dairy cattle using the unweighted UniFrac distance matrix. Value of *p* indicates a difference in beta diversity between sample types.

**Figure 2:**
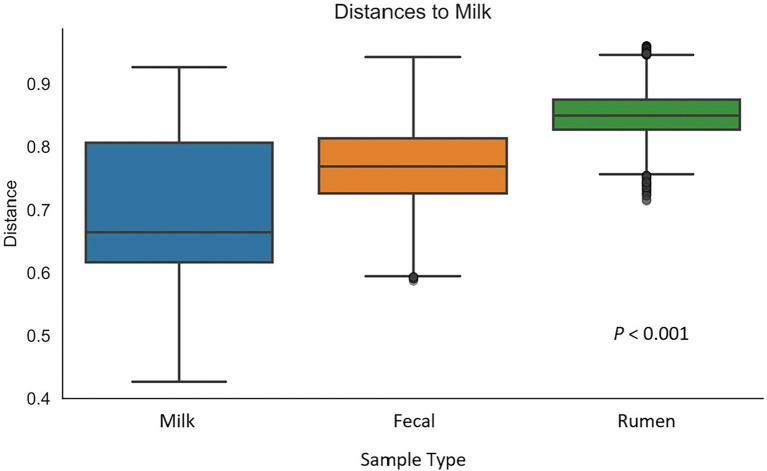
Box-and-whisker plot of unweighted UniFrac distances between milk samples (*n* = 47) and rumen (*n* = 51), and fecal (*n* = 51) samples. Whiskers express the maximum and minimum values. Value of *p* indicates a difference in diversity between sample types.

**Figure 3:**
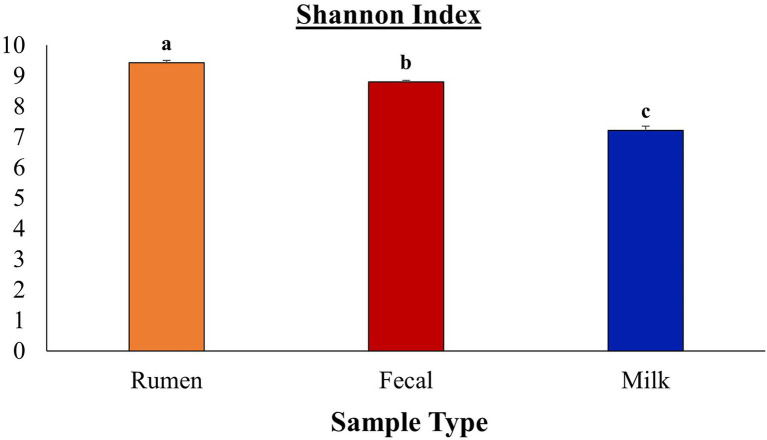
Comparison of Shannon diversity indices between rumen, fecal, and milk microbiota from Holstein dairy cattle. Sample types were significantly different (*p* ≤ 0.05) according to Bonferroni’s multiple comparisons.

After all the quality-filtering steps were applied, the resulting number of sequences in all samples ranged from 27,233 to 104,655 and were rarefied to a sequencing depth of 27,230 sequences per sample. A total of 40 phyla, 320 families, 733 genera, and 377 species were identified, with a number of other species not being identified by our classifier. The rumen, fecal, and milk microbiotas of the dairy cattle were very different (1,414, 987, 607 observed ASVs, respectively). Beta diversity between all pairs of samples was calculated using unweighted UniFrac distances. Rumen, fecal, and milk samples were all significantly diverse from each other (*p* < 0.001; Figure 1). Milk samples were not as clustered in comparison to rumen and fecal samples, indicating that the diversity between milk samples is more spread out than in ruminal or fecal samples. A box-and-whisker plot of the unweighted UniFrac distances revealed that the rumen population was farther in distance from the milk population in comparison to the fecal population (Figure 2), Supplementary Table 3 suggesting the fecal and milk populations may be more related than the milk and rumen populations. Shannon index (Figure 3) was similar to the beta-diversity findings and further demonstrated that the ruminal, fecal, and milk microbiotas were significantly distinct. This index revealed that microbial diversity was greatest (*p* < 0.001) in ruminal microbiota compared with the fecal microbiota and milk microbiota of dairy cows, reiterating how complex the rumen environment is. A greater ruminal microbial diversity compared to the feces was reported and suggested to be due to nutrient availability in the rumen, where most nutrients in feedstuffs are absorbed before they reach the large intestine (Siciliano-Jones and Murphy, 1989; Lourenco et al., 2020). It is not surprising that milk microbiota samples were the least diverse, as milk contains far less bacterial DNA than rumen or fecal samples (Dill-McFarland et al., 2017).

Table 1 summarizes the microbial richness and diversity metrics calculated for milk samples collected based on SCC values enumerated at the time of collection. Supplementary Table 4 summarizes the metrics based on daily milk yield. There were no differences in milk samples when the diversity metrics were calculated based on milk yield. However, when looking at SCC, the Shannon index was lower in cows with a high SCC in comparison to those with medium or low SCC values (*p* < 0.001). Milk samples from cows that had low SCC values were more evenly distributed in comparison to samples from cows with high SCC values (*p* = 0.053). The greater evenness observed in the milk microbiome of cows with low SCC values may be indicative that high SCC values contribute to a reduction in microbial evenness potentially due to (a) the influx of white blood cells that non-specifically kill and digest bacteria or (b) presence of infecting pathogens that either directly modify the microbiota through competitive means or indirectly through depletion of nutrients utilized by host microbiota, generating important differences between the microbiota of mastitic and non-mastitic cows. Differences could be due to pathogen abundance being increased in mastitic milk populations, how the infecting pathogen affects the abundance of non-pathogenic bacteria in normal milk populations (direct competition or predation), or how pathogens change the nutritional composition of the milk (e.g., competition for iron). It was suggested that pathogens may suppress regrowth of commensal bacteria, resulting in detrimental effects on udder homeostasis (Derakhshani et al., 2018). Cows identified to have a low SCC had more observed ASVs than cows with medium and high SCC values, although the difference was not significant. Rodrigues et al. (2017) found that bulk tank milk diversity was negatively correlated with bacterial richness, suggesting that the milk microbiomes from cattle with a high bacterial load are dominated by narrow groups of bacterial taxa. Furthermore, healthy quarters with a low milk SCC demonstrated a more diverse milk microbiome population than quarters with mastitis (Kuehn et al., 2013; Oikonomou et al., 2014; Metzger et al., 2018b). Present results demonstrated a lower (*p* < 0.001) Shannon diversity level as SCC levels increased, in agreement with previous studies.

**Table 1:** Alpha-diversity indices calculated for the milk samples of Holstein dairy cattle with different somatic cell count (SCC) ranges at time of collection: low, medium, or high.

Item	SCC range^1^			
	Low	Medium	High	Value of *p*
OBS_features^2^	581.37	571.27	490.92	0.061
Faith’s PD^3^	50.08	49.00	52.39	1
Shannon index^*^	7.809^a^	7.335^b^	5.909^c^	<0.001
Evenness^*^	0.8574^a^	0.8214^b^	0.6657^c^	<0.001

^a–c^Superscripts indicate difference (*p* ≤ 0.05) using Bonferroni’s pairwise comparisons.

1 Low: SCC ≤ 200,000 cells/ml. Medium: 200,000 cells/ml < SCC < 800,000 cells/ml. High: SCC *≥* 800,000 cells/mL.

2 Observed features.

3 Faith’s Phylogenetic Diversity.

* Means within row differed (*p* ≤ 0.05) according to Bonferroni’s multiple comparisons.

In contrast to milk, rumen samples revealed no differences (*p* > 0.05) for any diversity metrics calculated based on SCC (Table 2). Thus, although there were differences in milk microbiota based on SCC range, this is not reflected in the ruminal samples of the dairy cattle. Past research found a significant shift in rumen microbiota associated with inflammation and immune responses during mastitis, and a decrease in diversity of rumen microbiota in cows with mastitis when compared to healthy cows (Wang et al., 2021). While an association of SCC and rumen microbiota may not be present in the current study, further investigation of differing SCC and IMI status with rumen diversity is necessary. Similar to milk samples, ruminal samples revealed no differences for these diversity metrics when calculated based on milk yield (Supplementary Table 5).

**Table 2:** Alpha-diversity indices calculated for the rumen samples of Holstein dairy cattle with different somatic cell count (SCC) ranges at time of collection: low, medium, or high.

Item	SCC range^1^			
	Low	Medium	High	Value of *p*
OBS_features^2^	1371.57	1522.45	1413.08	1
Faith’s PD^3^	60.47	64.88	61.58	0.756
Shannon index	9.365	9.628	9.364	1
Evenness	0.9005	0.9113	0.8958	1

1 Low: SCC ≤ 200,000 cells/ml. Medium: 200,000 cells/ml < SCC < 800,000 cells/ml. High: SCC ≥ 800,000 cells/ml.

2 Observed features.

3 Faith’s Phylogenetic Diversity. None of the means within each row were significantly different (*p* ≤ 0.05) according to Bonferroni’s multiple comparisons.

Fecal samples demonstrated that high SCC cows had a more evenly distributed fecal microbiota in comparison to low and medium SCC cows (*p* = 0.016; Table 3). In addition, there was a trend towards high SCC cows also having a more diverse fecal microbiome in comparison to medium and low SCC cows (*p* = 0.087). This was the opposite of what observed in milk samples and suggests while the milk microbiota became less diverse and had lower evenness during IMI or immune cell influx, the hindgut microbiota was more diverse and more evenly distributed. Though conjecture, perhaps the local immune response at the mucosal surface of the mammary gland reduces the mucosal immune response in the hindgut, allowing proliferation of different populations of commensal bacteria that are normally restricted in proliferation. Just as in both milk and rumen samples, there was no differences in fecal sample diversity indices when calculated based on milk yield (Supplementary Table 6).

**Table 3:** Alpha-diversity indices calculated for the fecal samples of Holstein dairy cattle with different somatic cell count ranges (SCC) at time of collection: low, medium, or high.

Item	SCC range^1^			
	Low	Medium	High	Value of *p*
OBS_features^2^	985.68	962.09	1,012	1
Faith’s PD^3^	39.77	39.20	40.59	1
Shannon index	8.753	8.749	8.970	0.087
Evenness^*^	0.8809^b^	0.8835^b^	0.9019^a^	0.016

^a–c^Superscripts indicate difference (*p* ≤ 0.05) using Bonferroni’s pairwise comparisons.

1 Low: SCC ≤ 200,000 cells/ml. Medium: 200,000 cells/ml < SCC < 800,000 cells/ml. High: SCC ≥ 800,000 cells/ml.

2 Observed features.

3 Faith’s Phylogenetic Diversity.

* Means within row differed (*p* ≤ 0.05) according to Bonferroni’s multiple comparisons;

In the rumen, more efficient animals have lower bacterial diversity and richness, while the opposite occurred in the large intestinal environment, with a higher bacterial richness and diversity correlated with a higher feed efficiency (De Oliveira et al., 2013; Shabat et al., 2016; Welch et al., 2020). With further investigation, use of these same diversity metrics to predict if cows are at a greater risk for developing IMI is possible (Metzger et al., 2018a,b,c). It was suggested that the incidence of mastitis is associated with altered composition and decreased diversity of intramammary microbiota, although whether this is a cause or effect is still to be determined (Oikonomou et al., 2014; Derakhshani et al., 2018). Currently, few studies have investigated milk microbiome richness and diversity in association with individual cow factors such as SCC (Metzger et al., 2018a,b,c; Scarsella et al., 2021) that did not solely involve bulk tank milk sampling. Scarsella et al. (2021) investigated the association of the udder IMI status with fecal and blood microbiomes, but similar to the current study lacking associations between SCC and rumen microbiomes, diversity of these populations was not associated with SCC levels. However, Scarsella et al. (2021) also did not find associations between SCC and fecal microbiota, in contrast to the current finding of high SCC cows demonstrating more evenly distributed fecal microbiota.

### Bacterial abundance at the phylum level

**Figure 4:**
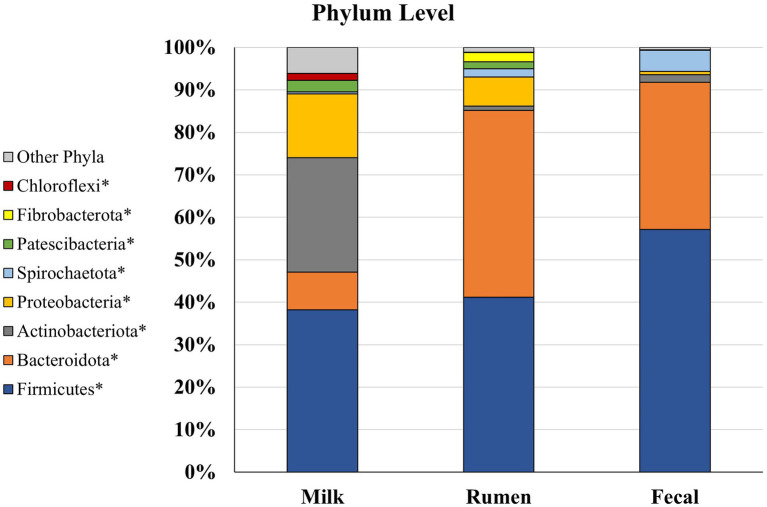
Relative bacterial abundance at the phylum level for Holstein dairy cattle in rumen (*n* = 51), fecal (*n* = 51), and milk samples (*n* = 47): phyla with relative abundance ≥1% in one of the sample groups. ^*^Samples within a phylum differ (*p* ≤ 0.05) according to Bonferroni’s multiple comparisons.

Of the eight main phyla comprising the microbiota of dairy cattle, four had significantly different (*p* < 0.05; Figure 4) abundances between the rumen, feces, and milk: Bacteroidota, Patescibacteria, Proteobacteria, and Spirochaetota. In the rumen, Fibrobacterota was detected at a higher abundance (*p* < 0.001) when compared to fecal and milk samples, whereas Firmicutes were found at greater abundance (*p* < 0.001) in feces when compared to rumen and milk samples. In previous studies investigating ruminal microbiota, Bacteroidota was the most prevalent phylum, followed by Firmicutes in several studies involving Angus beef calves (Lourenco et al., 2020), Brahman bulls (McCann et al., 2014), and a Brazilian Nelore steer (De Oliveira et al., 2013). In contrast, Firmicutes composed a greater amount of the microbial population in the feces, and was followed by Bacteroidota (De Oliveira et al., 2013). Members of the Bacteroidota phylum have many functions in the GIT, including degradation of carbohydrates such as complex plant cell walls and production of butyrate, a significant player in energy metabolism in the rumen (Thomas et al., 2011; Miguel et al., 2019). Members of the Firmicutes phylum serve an important role in the degradation of fiber and starch, and many produce butyrate, which is linked with gut health (Kim et al., 2011). Firmicutes has been denoted as a critical component for the milk microbiota, and it was the most predominant phyla in milk in the current study. While their specific role still has not been determined, Gram-positive Firmicutes were previously considered as contagious mastitis pathogens (Bhatt et al., 2012; Oikonomou et al., 2014; Rodrigues et al., 2017), though it is unclear whether this is the case in the present work. In contrast, some members of the phyla Firmicutes family, such as Lactobacillus are lauded as important dairy-related microbes, especially in fermented dairy products like yogurt (Widyastuti et al., 2021), and are considered beneficial probiotics. While the ratio of phyla Firmicutes to Bacteroidota in the rumen has been correlated to milk fat yield (Jami et al., 2014), little is known about the role of Bacteroidota in the milk microbiome. Bacteroidota was in much smaller abundance in the milk microbiome and did not compose any of the top 3 most abundant phyla in milk, which were Firmicutes, Actinobacteriota, and Proteobacteria. Proteobacteria consists of a wide variety of Gram-negative species that are considered environmental mastitis pathogens (Hogan et al., 1999; Bhatt et al., 2012), although the diversity within this phylum is large and includes non-pathogenic bacteria as well. Actinobacteriota includes Gram-positive bacteria that are regularly found in the rumen, although more investigation is needed on the ecology of this phylum in the rumen and how it may influence IMI in dairy cattle (Šuľák et al., 2012). Interestingly, another well-known pro-biotic Bifidobacterium is a member of phyla Actinobacteriota. Phylum Actinobacteriota was present in milk microbiota at a greater abundance (*p* < 0.05) than rumen and fecal microbiota. Chloroflexi contains bacteria with a diversity of roles, including aerobic thermophiles who use oxygen for growth and anoxygenic phototrophs who use light for photosynthesis (Ward et al., 2018), but the roles these members play in the gastrointestinal and milk microbiota remain unknown. Phylum Cyanobacteria was present in all three sample types, but no differences between populations were found (*p* > 0.05). Cyanobacteria consists of Gram-negative bacteria that share similarities to eukaryotic algae and includes toxin-producing bacteria that may cause disease in livestock, such as blue-green algae toxicosis (Puschner et al., 1998; McGorum et al., 2015). More investigation is needed to determine the impacts of this phylum on animal welfare in terms of production. Both Cyanobacteria and Chloroflexi have been identified before in milk samples but in low levels (Verdier-Metz et al., 2012; Ganda et al., 2016; Rodrigues et al., 2017). Phylum Chloroflexi was only present in milk microbiota, while Fibrobacterota was the only phylum present in rumen that was not identified in milk or fecal microbiota. Fibrobacterota has been previously identified as a core rumen bacterial taxon in dairy cows and suggested to be due to a higher forage-to-concentrate ratio (Xue et al., 2018), similarly used on the current study’s farm. Fecal microbiota did not have any phyla in abundance that were not found in rumen or milk populations suggesting that crosstalk exists between the rumen and fecal compartments, and potentially between the fecal and milk microbial populations.

Rumen and milk populations both had presence of phyla Proteobacteria and Patescibacteria that were not identified in the fecal microbiota potentially supporting the entero-mammary theory of how rumen microbiota is identified in milk (Rodríguez, 2014; Hu et al., 2022). Patescibacteria have been found to be prevalent in groundwater, sediment, and a variety of anoxic environments, but this phylum has not been well characterized in cattle microbiomes (Tian et al., 2020; Park et al., 2021). Overall, it is unsurprising to find some similarities in phyla identified in rumen, fecal, and milk communities, as bacteria from the rumen end up in the feces, on the exterior of the animal, and in the surrounding environment the animal is housed in Taponen et al. (2019).

Firmicutes, Proteobacteria, Bacteroidota, and Actinobacteriota have been identified as the most abundant phyla in milk (Verdier-Metz et al., 2012; Quigley et al., 2013; Ganda et al., 2016; Bonsaglia et al., 2017; Rodrigues et al., 2017; Derakhshani et al., 2018; Mary et al., 2022). Although, Cyanobacteria has also been identified as one of the most prevalent phyla present in milk samples over populations of Actinobacteriota and Bacteroidota (Bhatt et al., 2012; Rodrigues et al., 2017). Previous studies have indicated that the composition of bacterial communities in milk samples can differ between cows kept on different beddings and in different geographical locations, even with the milk is collected directly from the gland cistern, which may explain differences in microbiome populations of cows housed in different environments (Derakhshani et al., 2018; Metzger et al., 2018a,b,c; Taponen et al., 2019). Most importantly, the technique of collection from the mammary gland produces different microbiota results. For example, researchers have investigated collection of microbiome samples directly from the milk, using teat canal swabs, and on the teat apex (skin), and found that each location has their own bacterial taxa exclusive to their environment, as well as differing diversities and abundances (Andrews et al., 2019; Derakhshani et al., 2020). The results presented herein represent the milk microbiota, but potentially in addition the teat canal, mammary gland, and teat apex microbiota, as we cannot confirm all contaminants were removed with sanitizing steps. Moreover, environmental contamination cannot be ruled out. Comparison with other results must be performed carefully as the milk microbiota alone may not reflect the entirety of the bacterial population in the mammary gland.

### Bacterial abundance at the genus level

**Figure 5:**
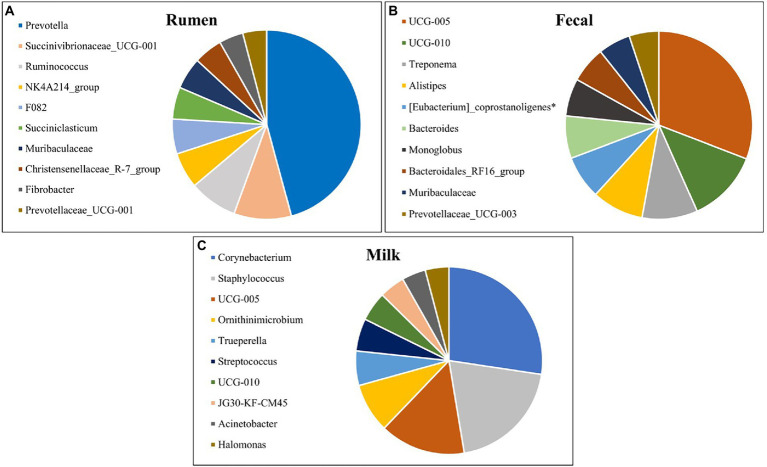
Top 10 most abundant bacterial genera detected in the **(A)** rumen (*n* = 51), **(B)** fecal (*n* = 51) and **(C)** milk (*n* = 47) samples of Holstein dairy cattle.

The top 10 most abundant genera for rumen, fecal, and milk populations of Holstein dairy cattle are shown in Figure 5. Rumen and fecal populations shared one genus amongst the 10 most abundant, *Muribaculaceae*, which was present in similar levels (2.85% vs 2.86%) in both environments. *Muribaculaceae*, formerly known as S24-7, is commonly found in the mammalian GIT (Lagkouvardos et al., 2019), and has been correlated with increased feed efficiency and increased intramuscular marbling in beef cattle (Krause et al., 2020; Welch et al., 2021), but its role in the milk microbiota is still unknown. Fecal and milk populations shared the genera *UCG-005* and *UCG-010*, both which were present in higher abundance in the fecal microbiota. *UCG-005* and *UCG-010* are from the Ruminococcaceae family under phylum Firmicutes and are obligate anaerobes that have been previously isolated in beef cattle with increased residual-feed intake (Welch et al., 2021). Interestingly, none of the most abundant genera were shared between the rumen and milk microbiotas. The presence of genera in milk samples that are usually found in fecal microbial populations and the absence of similar genera shared between ruminal and milk microbiomes suggest that the fecal microbiota may inform the milk population more than the ruminal microbiota.

The most prevalent genera previously identified in both milk samples and on the teat apex that were identified from milk samples in the present study include *Corynebacterium, Acinetobacter, Staphylococcus*, and *Streptococcus* (Vacheyrou et al., 2011; Bhatt et al., 2012; Braem et al., 2012; Verdier-Metz et al., 2012; Kuehn et al., 2013; Oikonomou et al., 2014; Ganda et al., 2016; Bonsaglia et al., 2017; Rodrigues et al., 2017; Derakhshani et al., 2018). *Corynebacterium*, identified as the most prevalent genera in milk in the present study, is frequently detected within cow’s milk and can assist in development of cheese flavor and aroma (Duthoit et al., 2003; Quigley et al., 2013; Oikonomou et al., 2014). However, one species of this genus, *Corynebacterium bovis*, is a well-known mastitis pathogen associated with decreased milk production (Watts et al., 2000). *Acinetobacter* is an important spoilage bacteria and can adapt to a variety of environmental conditions, but pathogens from this genus have been reported (Gurung et al., 2013; Rodrigues et al., 2017). While genera *Acinetobacter* and *Staphylococcus* have been frequently detected in raw milk from healthy cows, species from these genera can be involved with development of IMI in dairy cattle if favorable conditions for pathogen growth persist (Braem et al., 2011; Gonçalves et al., 2016; Bonsaglia et al., 2017). *Streptococcus* has been identified in both healthy and mastitic milk samples, and while many of these species are considered pathogenic in terms of IMI, some are frequently isolated in milk and are used as starter cultures in the manufacture of dairy products (Randazzo et al., 2002; Duthoit et al., 2003; Santarelli et al., 2008; Quigley et al., 2013; Oikonomou et al., 2014). *Halomonas* comprises Gram-negative aerobic bacteria under phylum Proteobacteria and is an abundant genus found in both milk and cheese (Coton et al., 2012; Pang et al., 2018), but the impact of these bacteria in terms of disease, if any, is still under investigation (Kim et al., 2013). *Trueperella pyogenes* is a common summer mastitis pathogen (Ishiyama et al., 2017), and was present in a single sample at high abundance, indicating a potential subclinical IMI. *Ornithinimicrobium* has been detected previously in milk and bedding microbiome samples (Dean et al., 2021; Li et al., 2021), and was linked to be significantly higher in sand bedding in comparison to other beddings (Ray et al., 2022), which is the type of bedding used in the present study.

### Comparing differing levels of milk yield with milk microbiota at the phylum level

The bacterial phyla detected in the milk samples of dairy cattle that were associated with milk yield are listed in Table 4. No bacterial phyla detected in rumen or fecal samples of dairy cattle were significantly associated with milk yield (data not shown). As correlations between hindgut microbiota and milk yield have been previously discovered (Xue et al., 2018), a link between these populations with milk production variables was expected. However, the present study indicates that the milk microbiota population may be more critical in terms of milk production than the hindgut populations. When examining the milk samples, cows with low and medium milk yield had higher levels of phylum Bacteroidota present in comparison to cows with high milk yield (*p* = 0.017). In addition, low milk yield cows had a trending abundance of phylum Elusimicrobiota in comparison to cows with medium and high milk yield (*p* = 0.076). Elusimicrobiota has been previously identified in the rumen of cattle, and while its role in animal health is still poorly understood, this phylum has been linked to dissemination of antimicrobial resistance genes (López-Catalina et al., 2021). More investigation is required to determine if these phyla in milk directly impact milk production, or if their changes in abundance in cows with different levels of milk production are due to a dilution/concentration effect.

**Table 4:** Bacterial phyla relative abundance (ppm) in the milk samples of Holstein dairy cattle associated with different milk yield ranges at time of collection: low, medium, and high.

Phyla	Milk yield range^1^			
	Low	Medium	High	Value of *p*
Abditibacteriota	53^b^	242^a^	0^b^	0.019
Bacteroidota	95,112^a^	91,862^a^	75,482^b^	0.017
Campilobacterota	241^b^	879^a^	710^a,b^	0.008
Deinococcota	2,864^b^	6,894^a^	5,762^a^	0.016
Elusimicrobiota	390	0	7	0.076
Fibrobacterota	52^b^	678^a^	121^b^	<0.001
Fusobacteriota	643^b^	1,881^a^	2,022^a^	<0.001
Gemmatimonadota	1,224	2,908	2,271	0.059
Halobacterota	16	0	241	0.067
Myxococcota	77^c^	605^b^	1,072^a^	0.004
Synergistota	30^b^	308^a^	104^b^	<0.001
Verrucomicrobiota	3,352^a,b^	3,069^b^	4,121^a^	0.036

^a–c^Superscripts indicate difference (*p* ≤ 0.05) using Bonferroni’s pairwise comparisons.

1 Low: Yield ≤ 65 lbs/day. Medium: 65 lbs < Yield < 90 lbs. High: Yield ≥ 90 lbs/day. Means within each row differed (*p* ≤ 0.05) according to Bonferroni’s multiple comparisons.

Cows with medium milk yield had higher levels of phyla Abditibacteriota, Fibrobacterota, and Synergistota present in their milk samples in comparison to cows with low and high milk yield (*p* < 0.05). Abditibacteriota, formerly phylum FBP, has one member that has been isolated from soil samples suggesting that environmental exposure contributed to its presence (Tahon et al., 2018). Synergistota has been isolated from rumen samples of cows previously (Glendinning et al., 2021), and in humans, members of Synergistota are suggested to have both a functional role in microflora as a commensal as well as act as opportunistic pathogens (Marchandin et al., 2010). Both medium and high milk yield cows had higher levels of phyla Campilobacterota, Deinococcota, and Fusobacteriota when compared to cows with low milk yield (*p* < 0.05). Phylum Campilobacterota includes sulfur-oxidizing bacteria that are commensals of livestock, but also includes the most common foodborne pathogenic bacteria, *Campylobacter* spp., that cause human enteritis (Wesley et al., 2000). Deinococcota members have been isolated from animal feces and milk products before (Yu et al., 2017), but no literature is available on the role of this phylum in cattle health. Fusobacteriota contains Gram-negative bacteria that are normal microflorae in the rumen of cattle that assist in metabolizing lactic acid (Tadepalli et al., 2009). Furthermore, Fusobacterium necrophorum has an important role in development of liver abscesses and foot rot in beef cattle (Nagaraja et al., 2005).

Milk samples from high milk yield cows had higher levels of phyla Myxococcota and Verrucomicrobiota compared with samples from low and medium milk yield cows (*p* < 0.05). Additionally, high milk yield cows had a trending abundance of phylum Halobacterota in comparison to the other two groups (*p* = 0.067). Myxococcota members are commonly found in soil and sediments, and are among the least investigated groups of bacteria, especially in the context of host-associated environments (Jingjing et al., 2022). Verrucomicrobiota, mostly free-living bacteria found in water, soil, and mammalian gut environments, was previously identified to be in lower proportion in rumen samples from high milk yield cows (Zhang G. et al., 2019). The phylum Halobacterota includes many methanogens that generate methane as the final product of metabolism (Lyu et al., 2018). A positive correlation between milk yield and methane emission has been reported before (Garnsworthy et al., 2012), which may explain the trending abundance of Halobacterota in milk samples from cows with high milk yield.

### Comparing differing levels of SCC with milk microbiota at the phylum level

The bacterial phyla detected in the milk of dairy cattle that were associated with SCC are listed in Table 5. No bacterial phyla detected in rumen or fecal samples of dairy cattle were associated with SCC. Previously, the relative abundance of phyla Actinobacteria and SR1 in the rumen were different among cows with different SCC levels; however, the predominant bacterial groups in the rumen did not vary (Zhong et al., 2018). While the gut microbiota likely play a role in the development of mastitis (Hu et al., 2022), these populations may not have a direct impact on mammary health indicators like SCC.

**Table 5:** Bacterial phyla relative abundance (ppm) in the milk samples of Holstein dairy cattle associated with different somatic cell count (SCC) ranges at time of collection: low, medium, and high.

Phyla	SCC range^1^			
	Low	Medium	High	Value of *p*
Abditibacteriota	221^a^	0^b^	15^b^	0.007
Bacteroidota	96,482	78,302	77,826	0.090
Chloroflexi	18,197^a^	12,183^b^	14,384^b^	0.029
Deinococcota	6,324^a^	6,445^a^	3,000^b^	0.007
Myxococcota	528	247	1,144	0.080
Patescibacteria	29,519^a^	32,565^a^	15,414^b^	0.006
Proteobacteria	149,794^b^	201,157^a^	99,111^c^	<0.001
Spirochaetota	5,930^a^	2,021^b^	3,737^b^	0.010

^a–c^Superscripts indicate difference (*p* ≤ 0.05) using Bonferroni’s pairwise comparisons.

1 Low: SCC ≤ 200,000 cells/ml. Medium: 200,000 cells/ml < SCC < 800,000 cells/ml. High: SCC ≥ 800,000 cells/ml. Means within each row differed (*p* ≤ 0.05) according to Bonferroni’s multiple comparisons.

Interestingly, while high milk yield cows had higher levels of phylum Myxococcota in their milk samples compared to low and medium milk yield cows, high SCC cows had a trending abundance of this phylum in comparison to lower SCC groups (*p* = 0.08). Another notable finding was the abundance of phylum Proteobacteria in medium SCC cows in comparison to low and high SCC cows (*p* < 0.001). Since many species of this phylum are associated with IMI, abundance would also be expected in high SCC cows. This finding could indicate that some cows enrolled in the medium SCC group may have had subclinical IMI at the time of collection, potentially more than those enrolled in the high SCC group. Though some groups define subclinical IMI as SCC more than 200,000 cells/ml, which our medium SCC group would fall into, without positive culture results, an official subclinical diagnosis cannot be made. However, Proteobacteria was also more abundant in samples from low SCC cows than high SCC cows, suggesting that members of this phylum have a bigger role in promotion of mammary health, potentially greater than the negative effect caused by the opportunistic pathogens that belong to this group.

Cows with low and medium SCC values at the time of collection had higher levels of phyla Deinococcota and Patescibacteria present in their milk samples in comparison to cows with high SCC values (*p* < 0.05). While Patescibacteria has been previously identified in high abundance in rumen and fecal samples of dairy cattle (Sun et al., 2019; Zhao et al., 2022), little information about the role of this phylum in these microbiomes is available. Similarly, information on Deinococcota is absent in milk microbiome studies. However, in the current study Deinococcota abundance was linked to both low SCC cows and high milk yield cows, the two most desirable production and quality metrics in a dairy herd.

Cows with low SCC values at the time of collection had significantly higher levels of phyla Abditibacteriota, Chloroflexi, and Spirochaetota present in their samples in comparison to cows with medium and high SCC values (*p* < 0.05). More investigation is required to determine if these phyla in milk directly impact the immune response or basal levels of SCC, or if their changes in abundance in cows with different levels of SCC are due to a dilution/concentration effect. The presence of Chloroflexi in high abundance in low SCC cows may suggest members of this phylum can be beneficial to host health, immune responses, or basal SCC in the mammary gland. Spirochaetota has members that cause prevalent diseases in mammals (Collighan and Woodward, 1997). While a high prevalence of this phylum has been reported in bulk tank milk samples before (Rodrigues et al., 2017), no study has focused on its role in mammary health. The higher abundance of Spirochaetota in low SCC cows should be investigated further.

### Comparing differing levels of milk yield with milk microbiota at the genus level

The bacterial genera detected in the milk of dairy cattle that were associated with milk yield are listed in Table 6. No bacterial genera detected in rumen or fecal samples of dairy cattle were significantly associated with milk yield, similar to the hindgut microbiota at the phyla level. Milk samples from cows that had high milk yield at the time of collection had higher amounts of bacteria genera *Escherichia-Shigella, Hymenobacter*, and *Sphingobacterium* present in their milk samples compared to cows that had medium and low milk yield (*p* < 0.05). Some members of genus *Escherichia-Shigella* are pathogenic, such as *Escherichia coli*, a common mastitis pathogen that can affect high producing cows in dairy herds and in severe cases result in death (Burvenich et al., 2003; Yu et al., 2021). It is interesting that cows with high milk yield had significantly higher abundance of this genus, as it is expected that pathogenic bacteria in this genus would negatively impact milk production (Heikkilä et al., 2018), but results demonstrate it may be more complicated than previously thought. It could be that instead of a negative impact on milk production, that high-producing cows are more susceptible to infections by pathogens like *E. coli*. *Hymenobacter*, under phylum Actinobacteria, has been previously identified in the teat canal microbiota (Falentin et al., 2016). Additionally, *Sphingobacterium* prevalence in bovine milk was associated with increased SCC; however, a single sample with a high prevalence is what resulted in this positive correlation (Oikonomou et al., 2014). In contrast, Wang et al. (2020) found *Sphingobacterium* presence in milk samples to be negatively correlated with SCC, whereas in the current study, no significant association was found between *Sphingobacterium* and SCC.

**Table 6:** Bacterial genera relative abundance (ppm) in the milk samples of Holstein dairy cattle associated with different milk yield ranges at time of collection: low, medium, and high.

Genera	Milk yield range^1^			
	Low	Medium	High	Value of *p*
*Acinetobacter*	5,387^c^	17,445^a^	13,080^b^	<0.001
*Actinomyces*	546^c^	1,629^a^	988^b^	<0.001
*Bifidobacterium*	13,543^a^	4,585^c^	8,267^b^	0.003
*Escherichia-Shigella*	1,918^b^	1,345^b^	6,376^a^	<0.001
*Fusobacterium*	433^b^	1,261^a^	1,313^a^	0.001
*Hymenobacter*	92^b^	0^c^	212^a^	0.030
*Peptostreptococcus*	246^b^	887^a^	1,077^a^	0.043
*Sphingobacterium*	313^b^	335^b^	1,760^a^	<0.001
*UCG-005*	65,865^a^	39,699^b^	40,177^b^	0.008

^a–c^Superscripts indicate difference (*p* ≤ 0.05) using Bonferroni’s pairwise comparisons.

1 Low: Yield ≤ 65 lbs/day. Medium: 65 lbs < Yield < 90 lbs. High: Yield ≥ 90 lbs/day. Means within each row differed (*p* ≤ 0.05) according to Bonferroni’s multiple comparisons.

Milk samples from cows with both medium and high milk yield at the time of collection had higher levels of genera *Acinetobacter, Actinomyces, Fusobacterium*, and *Peptostreptococcus* (*p* < 0.05). *Acinetobacter* may be important for the health status of cows (Pang et al., 2018), and potentially play a role in milk production levels. *Actinomyces* consists of Gram-positive, anaerobic bacteria that are inhabitants of the bovine mouth, and can cause the disease actinomycosis (lumpy jaw) in cattle (Gillespie et al., 2017). It is possible that suckling either by a calf after calving or from herd mates early in life contributed to the introduction of *Actinomyces*, one of the theories as to how the milk microbiome is established. The genus *Fusobacterium* also consists of species that have been linked with disease in cattle, but only when displaced from their normal ruminal environment where they assist in metabolizing lactic acid and degrading feed (Tadepalli et al., 2009). *Peptostreptococcus* identified in milk samples was suggested to have a potential role in the development of mastitis (Schwaiger et al., 2012), but information is limited on other roles this genus may have.

Milk samples from cows that had low milk yield at the time of collection had higher amounts of bacteria genera *Bifidobacterium* and *UCG-005* present in their samples in comparison to milk samples from cows that had medium and high milk yield (*p* < 0.05). More investigation is warranted on *UCG-005*, as it was the third most abundant genera present in milk samples and was significantly associated with low milk yield. *Bifidobacterium* is one of the major genera that make up the GIT of mammals and have been utilized as cattle probiotics to improve health (Gavini et al., 2006; Lin et al., 2020). It is surprising to find an abundance of this genus in milk to be associated with cows with low milk yield, as it has been demonstrated in other work that *Bifidobacterium* presence in milk samples was positively correlated with milk yield (Wang et al., 2020).

### Comparing differing levels of SCC with milk microbiota at the genus level

The bacterial genera detected in the milk of dairy cattle that were associated with SCC are listed in Table 7. No bacterial genera detected in rumen or fecal samples of dairy cattle were significantly associated with SCC, similar to the phyla level. Cows with high SCC values at the time of collection had significantly higher levels of genus *Staphylococcus* present in milk samples in comparison to cows with low and medium SCC values (*p* = 0.002). It is understood that species of this genus are associated with IMI, and it is not without possibility that cows enrolled with high SCC values may have had subclinical IMI at the time of collection caused by species of this genus, such as *Staphylococcus aureus*. *Marinobacter* and *Thiopseudomonas* were identified to be associated with cows with medium SCC values at the time of collection, but information on these genera is limited in bovine microbiome research.

**Table 7:** Bacterial genera relative abundance (ppm) in the milk samples of Holstein dairy cattle associated with different somatic cell count (SCC) ranges at time of collection: low, medium, and high.

Genera	SCC range^1^			
	Low	Medium	High	Value of *p*
*Acholeplasma*	2,905^a^	3,032^a^	188^b^	0.005
*Aeromicrobium*	2,064^a^	1,441^a^	317^b^	0.001
*Alloiococcus*	8,151^a^	5,322^a,b^	3,089^b^	0.013
*Alloprevotella*	2,275^a^	908^b^	715^b^	0.010
*Anaerosporobacter*	3,248^a^	1,150^b^	1,616^b^	<0.001
*Atopostipes*	2,546^a^	2,560^a^	467^b^	<0.001
*Facklamia*	10,710^a^	13,178^a^	2,866^b^	0.028
*Halomonas*	14,340^a^	17,290^a^	5,897^b^	0.010
*Marinobacter*	11,917^b^	18,718^a^	4,675^c^	<0.001
*Nocardioides*	5,841^a^	4,671^b^	2,730^c^	<0.001
*Ornithinimicrobium*	31,974^a^	27,555^a^	15,594^b^	0.014
*Pseudomonas*	8,640^a^	10,711^a^	3,265^b^	0.032
*Ruminococcus*	6,263^a^	2,522^b^	2,009^b^	0.004
*Staphylococcus*	16,682^b^	33,581^b^	181,994^a^	0.002
*Thiopseudomonas*	2,480^b^	7,520^a^	307^c^	<0.001
*Tissierella*	1,778^b^	3,646^a^	200^c^	<0.001
*Treponema*	5,273^a^	1,410^b^	3,568^a,b^	0.013

^a–c^Superscripts indicate difference (*p* ≤ 0.05) using Bonferroni’s pairwise comparisons.

1 Low: SCC ≤ 200,000 cells/ml. Medium: 200,000 cells/ml < SCC < 800,000 cells/ml. High: SCC ≥ 800,000 cells/ml. Means within each row differed (*p* ≤ 0.05) according to Bonferroni’s multiple comparisons.

Milk samples from cows identified as having low and medium SCC values at the time of collection had higher levels of bacteria genera *Acholeplasma, Aeromicrobium, Atopostipes, Facklamia, Halomonas, Nocardioides, Pseudomonas*, and *Tissierella* in comparison to milk samples from cows identified as having high SCC values (*p* < 0.05). Species of the *Acholeplasma* genus are common nonpathogenic contaminants found in milk, however, have been previously isolated in mastitic milk as well (Boonyayatra et al., 2011). Researchers have identified a negative correlation between *Aeromicrobium* abundance in bovine milk and SCC (Wang et al., 2020); the current study further reiterates these findings. While the Gram-positive members of *Atopostipes* have been identified in cattle microbiomes in past studies as well, they have not been linked to mammary gland health (Pang et al., 2018). Little is known about the Gram-positive species of *Facklamia*, but these species can be rare etiological agents of human infection and have been identified in a urine sample from a lactating cow and a bulk tank raw milk sample (Takamatsu et al., 2006; Rahmati et al., 2017; Doll et al., 2021). While *Nocardioides* has been discovered in bovine milk samples before (Delbès et al., 2007), little information is available on their association with mammary health. In contrast, genus *Pseudomonas* is a known taxon in the dairy industry, as certain species are causative pathogens of bovine IMI and potentially increase SCC levels as a result of infection (Park et al., 2014). The association with low and medium SCC values in the current study is surprising, although previous studies have demonstrated that *Pseudomonas aeruginosa* IMI resulted in the lowest SCC levels in comparison to *Staph. aureus, E. coli*, and other pathogens (Sumon et al., 2020). Lastly, *Tissierella* is not well studied in cattle health, but the Gram-negative species of this genus can cause infections in humans and have been identified in bovine digital dermatitis legions along with *Fusobacterium* spp. (Caméléna et al., 2016; Khalil et al., 2022).

Potentially more importantly, cows with low SCC at the time of collection had significantly higher levels of *Alloprevotella, Anaerosporobacter*, and *Ruminococcus* present in their milk samples in comparison to cows with medium and high SCC values (*p* < 0.05), and higher levels of genus *Treponema* present when only compared to cows with medium SCC values (*p* = 0.013). *Alloprevotella* still remains uninvestigated in terms of the bovine microbiomes but has been identified in the mouse GIT and human oral cavity (Downes et al., 2013; Liu et al., 2021). *Anaerosporobacter* presence in bovine rumen and fecal contents was negatively related to cow age (Zhang T. et al., 2019). *Ruminococcus* is a well-known ruminal bacterial group in cattle, which serve to degrade complex carbohydrates such as forage (La Reau and Suen, 2018). *Ruminococcus* presence in the rumen is negatively correlated with milk production (Jami et al., 2014; Chuang et al., 2020), although the current study found no association between milk production and *Ruminococcus* abundance in milk. *Treponema*, falling under phylum Spirochaetota, includes bacteria associated with bovine digital dermatitis (Klitgaard et al., 2014; Khalil et al., 2022). Low SCC cows also had higher levels of genus *Ornithinimicrobium* and *Alloiococcus* present in comparison to high SCC cows, but little is known about the role of this genera in cattle.

## Conclusion

Collectively, our results demonstrated that the ruminal, fecal, and milk microbiotas of dairy cows were very distinct. Alpha and beta-diversities, and the individual microbial compositions at the phylum level agreed that the ruminal, fecal, and milk environments were dissimilar. However, some bacterial taxa were present in all the environments, suggesting that there may be some relationship or nexus between these compartments and potentially influencing how the milk microbiome is established. Many of the bacteria identified to be linked with SCC and milk yield have various roles, and their functions may differ depending on the compartment they reside in. Moreover, milk samples associated with higher SCC values decreased the evenness of the microbial population. Investigation of mastitis pathogens in association with the milk microbiome should be done to determine if pathogens directly or indirectly alter the milk microbiota. Additionally, further research is required to investigate the presence of a biological pathway between the rumen and fecal microbiota and the resident milk microbiota to determine if the milk microbiome is established through the GIT microbiomes.

## Data availability statement

The sequencing data is publicly available on the MG-RAST website (www.mg-rast.org) under accession number mgm4977322.3.

## Ethics statement

The animal study was reviewed and approved by University of Georgia Office of Animal Care and Use AUP #A2021 07-029-Y1-A0.

## Author contributions

JW wrote the manuscript with the help of all the authors. JW and JL performed data analysis. VR, JL, and TC revised the manuscript. All authors contributed to the article and approved the submitted version.

## Funding

This work was supported by the University of Georgia Office of Research through the Faculty Seed Grants in the Sciences.

## Conflict of interest

The authors declare that the research was conducted in the absence of any commercial or financial relationships that could be construed as a potential conflict of interest.

## Publisher’s note

All claims expressed in this article are solely those of the authors and do not necessarily represent those of their affiliated organizations, or those of the publisher, the editors and the reviewers. Any product that may be evaluated in this article, or claim that may be made by its manufacturer, is not guaranteed or endorsed by the publisher.

## References

[ref1] Addis M. F. Tanca A. Uzzau S. Oikonomou G. Bicalho R. C. Moroni P. (2016). The bovine milk microbiota: insights and perspectives from -omics studies. Mol. BioSyst. 12, 2359–2372. doi: 10.1039/C6MB00217J, PMID: 27216801

[ref2] Alhussien M. N. Dang A. K. (2018). Milk somatic cells, factors influencing their release, future prospects, and practical utility in dairy animals: an overview. Vet. World 11, 562–577. doi: 10.14202/vetworld.2018.562-577, PMID: 29915493 PMC5993762

[ref3] Andrews T. Neher D. A. Weicht T. R. Barlow J. W. (2019). Mammary microbiome of lactating organic dairy cows varies by time, tissue site, and infection status. PLoS One 14, –e0225001. doi: 10.1371/journal.pone.0225001, PMID: 31725757 PMC6855453

[ref4] Ballou L. U. Pasquini M. Bremel R. D. Everson T. Sommer D. (1995). Factors affecting herd milk composition and milk plasmin at four levels of somatic cell counts. J. Dairy Sci. 78, 2186–2195. doi: 10.3168/jds.S0022-0302(95)76846-1, PMID: 8598403

[ref5] Bhatt V. D. Ahir V. B. Koringa P. G. Jakhesara S. J. Rank D. N. Nauriyal D. S.. (2012). Milk microbiome signatures of subclinical mastitis-affected cattle analysed by shotgun sequencing. J. Appl. Microbiol. 112, 639–650. doi: 10.1111/j.1365-2672.2012.05244.x, PMID: 22277077

[ref02] Bolyen E. Rideout J. R. Dillon M. R. Bokulich N. A. Abnet C. C. Al-Ghalith G. A.. (2019). Reproducible, interactive, scalable and extensible microbiome data science using QIIME 2. Nat. Biotechnol. 37, 852–857. doi: 10.1038/s41587-019-0209-9 31341288 PMC7015180

[ref6] Bonsaglia E. C. R. Gomes M. S. Canisso I. F. Zhou Z. Lima S. F. Rall V. L. M.. (2017). Milk microbiome and bacterial load following dry cow therapy without antibiotics in dairy cows with healthy mammary gland. Sci. Rep. 7, 8067. doi: 10.1038/s41598-017-08790-5, PMID: 28808353 PMC5556035

[ref7] Boonyayatra S. Fox L. K. Gay J. M. Sawant A. Besser T. E. (2011). Discrimination between mycoplasma and Acholeplasma species of bovine origin using digitonin disc diffusion assay, nisin disc diffusion assay, and conventional polymerase chain reaction. J. Vet. Diagn. Investig. 24, 7–13. doi: 10.1177/1040638711425936, PMID: 22362930

[ref8] Braem G. De Vliegher S. Supré K. Haesebrouck F. Leroy F. De Vuyst L. (2011). (GTG)5-PCR fingerprinting for the classification and identification of coagulase-negative staphylococcus species from bovine milk and teat apices: a comparison of type strains and field isolates. Vet. Microbiol. 147, 67–74. doi: 10.1016/j.vetmic.2010.05.044, PMID: 20599332

[ref9] Braem G. De Vliegher S. Verbist B. Heyndrickx M. Leroy F. De Vuyst L. (2012). Culture-independent exploration of the teat apex microbiota of dairy cows reveals a wide bacterial species diversity. Vet. Microbiol. 157, 383–390. doi: 10.1016/j.vetmic.2011.12.031, PMID: 22266158

[ref10] Buitenhuis B. Lassen J. Noel S. J. Plichta D. R. Sørensen P. Difford G. F.. (2019). Impact of the rumen microbiome on milk fatty acid composition of Holstein cattle. Genet. Sel. Evol. 51, 23. doi: 10.1186/s12711-019-0464-8, PMID: 31142263 PMC6542034

[ref11] Burvenich C. Van Merris V. Mehrzad J. Diez-Fraile A. Duchateau L. (2003). Severity of *E. coli* mastitis is mainly determined by cow factors. Vet. Res. 34, 521–564. doi: 10.1051/vetres:2003023, PMID: 14556694

[ref01] Callahan B. J. McMurdie P. J. Rosen M. J. Han A. W. Johnson A. J. Holmes S. P.. (2016). DADA2: High-resolution sample inference from Illumina amplicon data. Nat. Mthods 13, 581–583. doi: 10.1038/nmeth.3869 PMC4927377 27214047

[ref12] Caméléna F. Pilmis B. Mollo B. Hadj A. Le Monnier A. Mizrahi A. (2016). Infections caused by *Tissierella praeacuta*: a report of two cases and literature review. Anaerobe 40, 15–17. doi: 10.1016/j.anaerobe.2016.04.015, PMID: 27112422

[ref13] Chuang S. T. Ho S. T. Tu P. W. Li K. Y. Kuo Y. L. Shiu J. S.. (2020). The rumen specific Bacteriome in dry dairy cows and its possible relationship with phenotypes. Animals 10, 1791. doi: 10.3390/ani10101791, PMID: 33019774 PMC7601041

[ref14] Collighan R. J. Woodward M. J. (1997). Spirochaetes and other bacterial species associated with bovine digital dermatitis. FEMS Microbiol. Lett. 156, 37–41. doi: 10.1111/j.1574-6968.1997.tb12702.x, PMID: 9368358

[ref15] Coton M. Delbés-Paus C. Irlinger F. Desmasures N. Le Fleche A. Stahl V.. (2012). Diversity and assessment of potential risk factors of gram-negative isolates associated with French cheeses. Food Microbiol. 29, 88–98. doi: 10.1016/j.fm.2011.08.020, PMID: 22029922

[ref16] De Haas Y. Barkema H. W. Veerkamp R. F. (2002). The effect of pathogen-specific clinical mastitis on the lactation curve for somatic cell count. J. Dairy Sci. 85, 1314–1323. doi: 10.3168/jds.S0022-0302(02)74196-9, PMID: 12086069

[ref17] De Oliveira M. N. V. Jewell K. A. Freitas F. S. Benjamin L. A. Tótola M. R. Borges A. C.. (2013). Characterizing the microbiota across the gastrointestinal tract of a Brazilian Nelore steer. Vet. Microbiol. 164, 307–314. doi: 10.1016/j.vetmic.2013.02.013, PMID: 23490556

[ref18] Dean C. J. Slizovskiy I. B. Crone K. K. Pfennig A. X. Heins B. J. Caixeta L. S.. (2021). Investigating the cow skin and teat canal microbiomes of the bovine udder using different sampling and sequencing approaches. J. Dairy Sci. 104, 644–661. doi: 10.3168/jds.2020-18277, PMID: 33131828

[ref19] Delbès C. Ali-Mandjee L. Montel M.-C. (2007). Monitoring bacterial communities in raw milk and cheese by culture-dependent and -independent 16S rRNA gene-based analyses. Appl. Environ. Microbiol. 73, 1882–1891. doi: 10.1128/AEM.01716-06, PMID: 17259356 PMC1828836

[ref20] Derakhshani H. Fehr K. B. Sepehri S. Francoz D. De Buck J. Barkema H. W.. (2018). Invited review: microbiota of the bovine udder: contributing factors and potential implications for udder health and mastitis susceptibility. J. Dairy Sci. 101, 10605–10625. doi: 10.3168/jds.2018-14860, PMID: 30292553

[ref21] Derakhshani H. Plaizier J. C. De Buck J. Barkema H. W. Khafipour E. (2020). Composition and co-occurrence patterns of the microbiota of different niches of the bovine mammary gland: potential associations with mastitis susceptibility, udder inflammation, and teat-end hyperkeratosis. Anim. Microbiome 2, 11. doi: 10.1186/s42523-020-00028-6, PMID: 33499931 PMC7807822

[ref22] Dill-McFarland K. A. Breaker J. D. Suen G. (2017). Microbial succession in the gastrointestinal tract of dairy cows from 2 weeks to first lactation. Sci. Rep. 7, 40864. doi: 10.1038/srep40864, PMID: 28098248 PMC5241668

[ref23] Doll E. V. Staib L. Huptas C. Scherer S. Wenning M. (2021). *Facklamia lactis* sp. nov., isolated from raw milk. Int. J. Syst. Evol. Microbiol. 71, 4869. doi: 10.1099/ijsem.0.004869, PMID: 34252020

[ref24] Dowd S. E. Callaway T. R. Wolcott R. D. Sun Y. McKeehan T. Hagevoort R. G.. (2008). Evaluation of the bacterial diversity in the feces of cattle using 16S rDNA bacterial tag-encoded FLX amplicon pyrosequencing (bTEFAP). BMC Microbiol. 8, 125. doi: 10.1186/1471-2180-8-125, PMID: 18652685 PMC2515157

[ref25] Downes J. Dewhirst F. E. Tanner A. C. R. Wade W. G. (2013). Description of Alloprevotella rava gen. nov., sp. nov., isolated from the human oral cavity, and reclassification of Prevotella tannerae Moore et al. 1994 as Alloprevotella tannerae gen. nov., comb. nov. Int. J. Syst. Evol. Microbiol. 63, 1214–1218. doi: 10.1099/ijs.0.041376-0, PMID: 22753527 PMC3709537

[ref26] Doyle C. J. Gleeson D. O'Toole P. W. Cotter P. D. (2016). Impacts of seasonal housing and teat preparation on raw milk microbiota: a high-throughput sequencing study. Appl. Environ. Microbiol. 83:e02694-16. doi: 10.1128/AEM.02694-16 27815277 PMC5203630

[ref27] Duthoit F. Godon J.-J. Montel M.-C. (2003). Bacterial community dynamics during production of registered designation of origin Salers cheese as evaluated by 16S rRNA gene single-strand conformation polymorphism analysis. Appl. Environ. Microbiol. 69, 3840–3848. doi: 10.1128/AEM.69.7.3840-3848.2003, PMID: 12839752 PMC165180

[ref28] Falentin H. Rault L. Nicolas A. Bouchard D. S. Lassalas J. Lamberton P.. (2016). Bovine teat microbiome analysis revealed reduced alpha diversity and significant changes in taxonomic profiles in quarters with a history of mastitis. Front. Microbiol. 7:480. doi: 10.3389/fmicb.2016.00480, PMID: 27242672 PMC4876361

[ref29] Ganda E. K. Bisinotto R. S. Lima S. F. Kronauer K. Decter D. H. Oikonomou G.. (2016). Longitudinal metagenomic profiling of bovine milk to assess the impact of intramammary treatment using a third-generation cephalosporin. Sci. Rep. 6, 37565. doi: 10.1038/srep37565, PMID: 27874095 PMC5118806

[ref30] Garnsworthy P. C. Craigon J. Hernandez-Medrano J. H. Saunders N. (2012). Variation among individual dairy cows in methane measurements made on farm during milking. J. Dairy Sci. 95, 3181–3189. doi: 10.3168/jds.2011-4606, PMID: 22612953

[ref31] Gavini F. Delcenserie V. Kopeinig K. Pollinger S. Beerens H. Bonaparte C.. (2006). Bifidobacterium species isolated from animal feces and from beef and pork meat. J. Food Prot. 69, 871–877. doi: 10.4315/0362-028X-69.4.871, PMID: 16629032

[ref32] Gillespie A. Bortolami A. Crosby-Durrani H. Verin R. (2017). An unusual presentation of actinomycosis in a dairy cow. Vet. Rec. Case Rep. 5:e000395. doi: 10.1136/vetreccr-2016-000395

[ref33] Glendinning L. Genç B. Wallace R. J. Watson M. (2021). Metagenomic analysis of the cow, sheep, reindeer and red deer rumen. Sci. Rep. 11, 1990. doi: 10.1038/s41598-021-81668-9, PMID: 33479378 PMC7820578

[ref34] Gonçalves J. L. Tomazi T. Barreiro J. R. Beuron D. C. Arcari M. A. Lee S. H. I.. (2016). Effects of bovine subclinical mastitis caused by *Corynebacterium* spp. on somatic cell count, milk yield and composition by comparing contralateral quarters. Vet. J. 209, 87–92. doi: 10.1016/j.tvjl.2015.08.009, PMID: 26831159

[ref35] Gurung M. Nam H. M. Tamang M. D. Chae M. H. Jang G. C. Jung S. C.. (2013). Prevalence and antimicrobial susceptibility of Acinetobacter from raw bulk tank milk in Korea. J. Dairy Sci. 96, 1997–2002. doi: 10.3168/jds.2012-5965, PMID: 23462164

[ref36] Hagey J. V. Bhatnagar S. Heguy J. M. Karle B. M. Price P. L. Meyer D.. (2019). Fecal microbial communities in a large representative cohort of California dairy cows. Front. Microbiol. 10:1093. doi: 10.3389/fmicb.2019.01093, PMID: 31156599 PMC6532609

[ref37] Heikkilä A.-M. Liski E. Pyörälä S. Taponen S. (2018). Pathogen-specific production losses in bovine mastitis. J. Dairy Sci. 101, 9493–9504. doi: 10.3168/jds.2018-14824, PMID: 30122416

[ref38] Hogan J. S. Gonzalez R. N. Harmon R. J. Nickerson S. C. Oliver S. P. Pankey J. W. (1999). Laboratory Handbook on Bovine Mastitis. National Mastitis Council Madison, WI, 78, 485–488.

[ref39] Hu X. Li S. Mu R. Guo J. Zhao C. Cao Y.. (2022). The rumen microbiota contributes to the development of mastitis in dairy cows. Microbiol. Spectr. 10:e0251221. doi: 10.1128/spectrum.02512-21, PMID: 35196821 PMC8865570

[ref40] Hungate R. E. (1966). The rumen and its microbes. Elsevier Inc., Academic Press.

[ref41] Ishiyama D. Mizomoto T. Ueda C. Takagi N. Shimizu N. Matsuura Y.. (2017). Factors affecting the incidence and outcome of *Trueperella pyogenes* mastitis in cows. J. Vet. Med. Sci. 79, 626–631. doi: 10.1292/jvms.16-0401, PMID: 28163273 PMC5383188

[ref42] Jami E. White B. A. Mizrahi I. (2014). Potential role of the bovine rumen microbiome in modulating milk composition and feed efficiency. PLoS One 9:e85423. doi: 10.1371/journal.pone.0085423, PMID: 24465556 PMC3899005

[ref43] Jingjing W. Jianing W. Shuge W. Zheng Z. Yuezhong L. (2022). Global geographic diversity and distribution of the Myxobacteria. Microbiol. Spectr. 9, e0001221. doi: 10.1128/Spectrum.00012-21, PMID: 34259548 PMC8552515

[ref44] John W. R. Goor S. C. G. P. Ilma T. Emma G. Paolo B. Pekka H. R. B. A.. (2022). A heritable subset of the core rumen microbiome dictates dairy cow productivity and emissions. Sci. Adv. 5, eaav8391. doi: 10.1126/sciadv.aav8391, PMID: 31281883 PMC6609165

[ref45] Khalil A. Batool A. Arif S. (2022). Healthy cattle microbiome and Dysbiosis in diseased phenotypes. Ruminants 2, 134–156. doi: 10.3390/ruminants2010009

[ref46] Kim D. Hofstaedter C. E. Zhao C. Mattei L. Tanes C. Clarke E.. (2017). Optimizing methods and dodging pitfalls in microbiome research. Microbiome 5, 52. doi: 10.1186/s40168-017-0267-5, PMID: 28476139 PMC5420141

[ref47] Kim K. K. Lee J.-S. Stevens D. A. (2013). Microbiology and epidemiology of Halomonas species. Future Microbiol. 8, 1559–1573. doi: 10.2217/fmb.13.108 24266356

[ref48] Kim M. Morrison M. Yu Z. (2011). Status of the phylogenetic diversity census of ruminal microbiomes. FEMS Microbiol. Ecol. 76, 49–63. doi: 10.1111/j.1574-6941.2010.01029.x, PMID: 21223325

[ref49] Klindworth A. Pruesse E. Schweer T. Peplies J. Quast C. Horn M.. (2013). Evaluation of general 16S ribosomal RNA gene PCR primers for classical and next-generation sequencing-based diversity studies. Nucleic Acids Res. 41:e1. doi: 10.1093/nar/gks808, PMID: 22933715 PMC3592464

[ref50] Klitgaard K. Nielsen M. W. Ingerslev H.-C. Boye M. Jensen T. K. (2014). Discovery of bovine digital dermatitis-associated *Treponema* spp. in the dairy herd environment by a targeted deep-sequencing approach. Appl. Environ. Microbiol. 80, 4427–4432. doi: 10.1128/AEM.00873-14, PMID: 24814794 PMC4068665

[ref51] Krause T. R. Lourenco J. M. Welch C. B. Rothrock M. J. Callaway T. R. Pringle T. D. (2020). The relationship between the rumen microbiome and carcass merit in Angus steers. J. Anim. Sci. 98. doi: 10.1093/jas/skaa287, PMID: 32877916 PMC7526868

[ref52] Kuehn J. S. Gorden P. J. Munro D. Rong R. Dong Q. Plummer P. J.. (2013). Bacterial community profiling of milk samples as a means to understand culture-negative bovine clinical mastitis. PLoS One 8:e61959. doi: 10.1371/journal.pone.0061959, PMID: 23634219 PMC3636265

[ref53] La Reau A. J. Suen G. (2018). The Ruminococci: key symbionts of the gut ecosystem. J. Microbiol. 56, 199–208. doi: 10.1007/s12275-018-8024-4, PMID: 29492877

[ref54] Lagkouvardos I. Lesker T. R. Hitch T. C. A. Gálvez E. J. C. Smit N. Neuhaus K.. (2019). Sequence and cultivation study of Muribaculaceae reveals novel species, host preference, and functional potential of this yet undescribed family. Microbiome 7, 28. doi: 10.1186/s40168-019-0637-2, PMID: 30782206 PMC6381624

[ref55] Li H. Wang X. Wu Y. Zhang D. Xu H. Xu H.. (2021). Relationships among bedding materials, bedding bacterial composition and lameness in dairy cows. Anim. Biosci. 34, 1559–1568. doi: 10.5713/ajas.20.0565, PMID: 33171032 PMC8495337

[ref56] Lin W.-C. Ptak C. P. Chang C.-Y. Ian M.-K. Chia M.-Y. Chen T.-H.. (2020). Autochthonous lactic acid bacteria isolated from dairy cow feces exhibiting promising probiotic properties and in vitro antibacterial activity Against foodborne pathogens in cattle. Front. Vet. Sci. 7:239. doi: 10.3389/fvets.2020.00239, PMID: 32500086 PMC7243249

[ref57] Liu Y. Cai J. Zhang F. (2021). Functional comparison of breast milk, cow milk and goat milk based on changes in the intestinal flora of mice. LWT 150:111976. doi: 10.1016/j.lwt.2021.111976

[ref58] López-Catalina A. Atxaerandio R. García-Rodríguez A. Goiri I. Gutierrez-Rivas M. Jiménez-Montero J. A.. (2021). Characterisation of the rumen resistome in Spanish dairy cattle. Anim. Microbiome 3, 63. doi: 10.1186/s42523-021-00125-0, PMID: 34551823 PMC8456196

[ref59] Lourenco J. M. Callaway T. R. Kieran T. J. Glenn T. C. McCann J. C. Stewart R. L. (2019). Analysis of the rumen microbiota of beef calves supplemented During the suckling phase. Front. Microbiol. 10:1131. doi: 10.3389/fmicb.2019.01131, PMID: 31191476 PMC6547912

[ref60] Lourenco J. M. Kieran T. J. Seidel D. S. Glenn T. C. Da Silveira M. F. Callaway T. R.. (2020). Comparison of the ruminal and fecal microbiotas in beef calves supplemented or not with concentrate. PLoS One 15, –e0231533. doi: 10.1371/journal.pone.0231533, PMID: 32282837 PMC7153887

[ref61] Lyu Z. Shao N. Akinyemi T. Whitman W. B. (2018). Methanogenesis. Curr. Biol. 28, R727–R732. doi: 10.1016/j.cub.2018.05.021 29990451

[ref62] Ma C. Sun Z. Zeng B. Huang S. Zhao J. Zhang Y.. (2018). Cow-to-mouse fecal transplantations suggest intestinal microbiome as one cause of mastitis. Microbiome 6, 200. doi: 10.1186/s40168-018-0578-1, PMID: 30409169 PMC6225715

[ref63] Marchandin H. Damay A. Roudière L. Teyssier C. Zorgniotti I. Dechaud H.. (2010). Phylogeny, diversity and host specialization in the phylum Synergistetes with emphasis on strains and clones of human origin. Res. Microbiol. 161, 91–100. doi: 10.1016/j.resmic.2009.12.008, PMID: 20079831

[ref64] Mary E. K. Yanin S. Miles L. Jose Z. Jeremy M. Jessie H.. (2022). The Core and seasonal microbiota of raw bovine milk in tanker trucks and the impact of transfer to a milk processing facility. MBio 7, e00836–e00816. doi: 10.1128/mBio.00836-16, PMID: 27555305 PMC4999540

[ref65] Matthews C. Crispie F. Lewis E. Reid M. O’Toole P. W. Cotter P. D. (2019). The rumen microbiome: a crucial consideration when optimising milk and meat production and nitrogen utilisation efficiency. Gut Microbes 10, 115–132. doi: 10.1080/19490976.2018.1505176, PMID: 30207838 PMC6546327

[ref66] McCann J. C. Wiley L. M. Forbes T. D. Rouquette F. M. Jr. Tedeschi L. O. (2014). Relationship between the rumen microbiome and residual feed intake-efficiency of Brahman bulls stocked on Bermudagrass pastures. PLoS One 9:e91864. doi: 10.1371/journal.pone.0091864, PMID: 24642871 PMC3958397

[ref67] McGorum B. C. Pirie R. S. Glendinning L. McLachlan G. Metcalf J. S. Banack S. A.. (2015). Grazing livestock are exposed to terrestrial cyanobacteria. Vet. Res. 46, 16. doi: 10.1186/s13567-015-0143-x, PMID: 25828258 PMC4342207

[ref68] Metzger S. A. Hernandez L. L. Skarlupka J. H. Suen G. Walker T. M. Ruegg P. L. (2018a). Influence of sampling technique and bedding type on the milk microbiota: results of a pilot study. J. Dairy Sci. 101, 6346–6356. doi: 10.3168/jds.2017-14212, PMID: 29680645

[ref69] Metzger S. A. Hernandez L. L. Skarlupka J. H. Walker T. M. Suen G. Ruegg P. L. (2018b). A cohort study of the milk microbiota of healthy and inflamed bovine mammary glands from Dryoff Through 150 days in milk. Front. Vete. Sci. 5, 247. doi: 10.3389/fvets.2018.00247, PMID: 30356776 PMC6189514

[ref70] Metzger S. A. Hernandez L. L. Suen G. Ruegg P. L. (2018c). Understanding the milk microbiota. Vet. Clin. N. Am. Food Anim. Pract. 34, 427–438. doi: 10.1016/j.cvfa.2018.06.003 30316501

[ref71] Miguel M. Lee S. S. Mamuad L. Choi Y. J. Jeong C. D. Son A.. (2019). Enhancing butyrate production, Ruminal fermentation and microbial population through supplementation with clostridium saccharobutylicum. J. Microbiol. Biotechnol. 29, 1083–1095. doi: 10.4014/jmb.1905.05016, PMID: 31216841

[ref72] Mu Y. Lin X. Wang Z. Hou Q. Wang Y. Hu Z. (2019). High-production dairy cattle exhibit different rumen and fecal bacterial community and rumen metabolite profile than low-production cattle. Microbiol. Open 8, –e00673. doi: 10.1002/mbo3.673, PMID: 30277648 PMC6460281

[ref73] Nagaraja T. G. Narayanan S. K. Stewart G. C. Chengappa M. M. (2005). Fusobacterium necrophorum infections in animals: pathogenesis and pathogenic mechanisms. Anaerobe 11, 239–246. doi: 10.1016/j.anaerobe.2005.01.007, PMID: 16701574

[ref74] O’Hara E. Neves A. L. Song Y. Guan L. (2020). The role of the gut microbiome in cattle production and health: driver or passenger? Annu. Rev. Anim. Biosci. 8, 199–220. doi: 10.1146/annurev-animal-021419-083952 32069435

[ref75] Oikonomou G. Addis M. F. Chassard C. Nader-Macias M. E. F. Grant I. Delbès C.. (2020). Milk microbiota: what are we exactly talking about? Front. Microbiol. 11, 60. doi: 10.3389/fmicb.2020.00060, PMID: 32117107 PMC7034295

[ref76] Oikonomou G. Bicalho M. L. Meira E. Rossi R. E. Foditsch C. Machado V. S.. (2014). Microbiota of cow’s milk; distinguishing healthy, sub-clinically and clinically diseased quarters. PLoS One 9:e85904. doi: 10.1371/journal.pone.0085904, PMID: 24465777 PMC3896433

[ref77] Pang M. Xie X. Bao H. Sun L. He T. Zhao H.. (2018). Insights Into the bovine milk microbiota in dairy farms with different incidence rates of subclinical mastitis. Front. Microbiol. 9, 2379. doi: 10.3389/fmicb.2018.02379, PMID: 30459717 PMC6232673

[ref78] Park T. Cersosimo L. M. Li W. Radloff W. Zanton G. I. (2021). Pre-weaning Ruminal Administration of Differentially-Enriched, rumen-derived Inocula shaped rumen bacterial communities and co-occurrence networks of post-weaned dairy calves. Front. Microbiol. 12:625488. doi: 10.3389/fmicb.2021.625488, PMID: 33717013 PMC7952535

[ref79] Park H. R. Hong M. K. Hwang S. Y. Park Y. K. Kwon K. H. Yoon J. W.. (2014). Characterisation of *Pseudomonas aeruginosa* related to bovine mastitis. Acta Vet. Hung. 62, 1–12. doi: 10.1556/AVet.2013.054, PMID: 24334080

[ref03] Pedregosa F. Varoquaux G. Gramfort A. Michel V. Thirion B. Grisel O.. (2011). Scikit-learn: Machine learning in python. J. Mach. Learn. Res. 12, 2825–2830. doi: 10.5555/1953048.2078195

[ref80] Puschner B. Galey F. D. Johnson B. Dickie C. W. Vondy M. Francis T.. (1998). Blue-green algae toxicosis in cattle. J. Am. Vet. Med. Assoc. 213, 1605–1607. PMID: 9838962

[ref04] Quast C. Pruesse E. Yilmaz P. Gerken J. Schweer T. Yarza P.. (2013). The SILVA ribosomal RNA gene database project: improved data processing and web-based tools. Nucleic Acids Res. 41, D590–D596. doi: 10.1093/nar/gks1219 23193283 PMC3531112

[ref81] Quigley L. O’Sullivan O. Stanton C. Beresford T. P. Ross R. P. Fitzgerald G. F.. (2013). The complex microbiota of raw milk. FEMS Microbiol. Rev. 37, 664–698. doi: 10.1111/1574-6976.12030 23808865

[ref82] Rahmati E. Martin V. Wong D. Sattler F. Petterson J. Ward P.. (2017). Facklamia species as an Underrecognized pathogen. Open forum. Infect. Dis. 4, ofw272. doi: 10.1093/ofid/ofw272, PMID: 28480264 PMC5414014

[ref83] Randazzo C. L. Torriani S. Akkermans A. D. L. de Vos W. M. Vaughan E. E. (2002). Diversity, dynamics, and activity of bacterial communities during production of an artisanal Sicilian cheese as evaluated by 16S rRNA analysis. Appl. Environ. Microbiol. 68, 1882–1892. doi: 10.1128/AEM.68.4.1882-1892.2002, PMID: 11916708 PMC123848

[ref84] Ray T. Gaire T. N. Dean C. J. Rowe S. Godden S. M. Noyes N. R. (2022). The microbiome of common bedding materials before and after use on commercial dairy farms. Anim. Microbiome 4, 18. doi: 10.1186/s42523-022-00171-2, PMID: 35256016 PMC8900318

[ref85] Rodrigues M. X. Lima S. F. Canniatti-Brazaca S. G. Bicalho R. C. (2017). The microbiome of bulk tank milk: characterization and associations with somatic cell count and bacterial count. J. Dairy Sci. 100, 2536–2552. doi: 10.3168/jds.2016-11540, PMID: 28189327

[ref86] Rodríguez J. M. (2014). The origin of human milk bacteria: is there a bacterial entero-mammary pathway during late pregnancy and lactation? Adv. Nutr. 5, 779–784. doi: 10.3945/an.114.007229, PMID: 25398740 PMC4224214

[ref87] Salter S. J. Cox M. J. Turek E. M. Calus S. T. Cookson W. O. Moffatt M. F.. (2014). Reagent and laboratory contamination can critically impact sequence-based microbiome analyses. BMC Biol. 12, 87. doi: 10.1186/s12915-014-0087-z, PMID: 25387460 PMC4228153

[ref88] Santarelli M. Gatti M. Lazzi C. Bernini V. Zapparoli G. A. Neviani E. (2008). Whey starter for grana Padano cheese: effect of technological parameters on viability and composition of the microbial community. J. Dairy Sci. 91, 883–891. doi: 10.3168/jds.2007-0296, PMID: 18292243

[ref89] Sanz-Fernandez M. V. Daniel J.-B. Seymour D. J. Kvidera S. K. Bester Z. Doelman J.. (2020). Targeting the hindgut to improve health and performance in cattle. Animals 10, 1817. doi: 10.3390/ani10101817, PMID: 33036177 PMC7600859

[ref90] Scarsella E. Zecconi A. Cintio M. Stefanon B. (2021). Characterization of microbiome on feces, blood and milk in dairy cows with different milk leucocyte pattern. Animals 11, 1463. doi: 10.3390/ani11051463, PMID: 34069719 PMC8160755

[ref91] Schären M. Frahm J. Kersten S. Meyer U. Hummel J. Breves G.. (2018). Interrelations between the rumen microbiota and production, behavioral, rumen fermentation, metabolic, and immunological attributes of dairy cows. J. Dairy Sci. 101, 4615–4637. doi: 10.3168/jds.2017-13736, PMID: 29454699

[ref92] Schwaiger K. Wimmer M. Huber-Schlenstedt R. Fehlings K. Hölzel C. S. Bauer J. (2012). Hot topic: bovine milk samples yielding negative or nonspecific results in bacterial culturing; the possible role of PCR-single strand conformation polymorphism in mastitis diagnosis. J. Dairy Sci. 95, 98–101. doi: 10.3168/jds.2011-4700, PMID: 22192188

[ref93] Shabat S. K. Sasson G. Doron-Faigenboim A. Durman T. Yaacoby S. Berg Miller M. E.. (2016). Specific microbiome-dependent mechanisms underlie the energy harvest efficiency of ruminants. ISME J. 10, 2958–2972. doi: 10.1038/ismej.2016.62, PMID: 27152936 PMC5148187

[ref94] Siciliano-Jones J. Murphy M. R. (1989). Nutrient digestion in the large intestine as influenced by forage to concentrate ratio and forage physical form. J. Dairy Sci. 72, 471–484. doi: 10.3168/jds.S0022-0302(89)79129-3, PMID: 2703569

[ref96] Šuľák M. Sikorová L. Jankuvová J. Javorský P. Pristaš P. (2012). Variability of Actinobacteria, a minor component of rumen microflora. Folia Microbiol. 57, 351–353. doi: 10.1007/s12223-012-0140-7, PMID: 22528311

[ref97] Sumon S. M. M. R. Parvin M. S. Ehsan M. A. Islam M. T. (2020). Dynamics of somatic cell count and intramammary infection in lactating dairy cows. J. Adv. Vet. Anim. Res. 7, 314–319. doi: 10.5455/javar.2020.g423, PMID: 32607363 PMC7320813

[ref98] Sun Z. Yu Z. Wang B. (2019). Perilla frutescens leaf alters the rumen microbial community of lactating dairy cows. Microorganisms 7, 562. doi: 10.3390/microorganisms7110562, PMID: 31766265 PMC6921060

[ref99] Tadepalli S. Narayanan S. K. Stewart G. C. Chengappa M. M. Nagaraja T. G. (2009). Fusobacterium necrophorum: a ruminal bacterium that invades liver to cause abscesses in cattle. Anaerobe 15, 36–43. doi: 10.1016/j.anaerobe.2008.05.005, PMID: 18595747

[ref100] Tahon G. Tytgat B. Lebbe L. Carlier A. Willems A. (2018). Abditibacterium utsteinense sp. nov., the first cultivated member of candidate phylum FBP, isolated from ice-free Antarctic soil samples. Syst. Appl. Microbiol. 41, 279–290. doi: 10.1016/j.syapm.2018.01.009, PMID: 29475572

[ref101] Takamatsu D. Ide H. Osaki M. Sekizaki T. (2006). Identification of Facklamia sourekii from a lactating cow. J. Vet. Med. Sci. 68, 1225–1227. doi: 10.1292/jvms.68.1225, PMID: 17146186

[ref102] Taponen S. McGuinness D. Hiitiö H. Simojoki H. Zadoks R. Pyörälä S. (2019). Bovine milk microbiome: a more complex issue than expected. Vet. Res. 50, 44. doi: 10.1186/s13567-019-0662-y, PMID: 31171032 PMC6555717

[ref103] Thomas F. Hehemann J.-H. Rebuffet E. Czjzek M. Michel G. (2011). Environmental and gut Bacteroidetes: the food connection. Front. Microbiol. 2:93. doi: 10.3389/fmicb.2011.00093, PMID: 21747801 PMC3129010

[ref104] Tian R. Ning D. He Z. Zhang P. Spencer S. J. Gao S.. (2020). Small and mighty: adaptation of superphylum Patescibacteria to groundwater environment drives their genome simplicity. Microbiome 8, 51. doi: 10.1186/s40168-020-00825-w, PMID: 32252814 PMC7137472

[ref105] Vacheyrou M. Normand A.-C. Guyot P. Cassagne C. Piarroux R. Bouton Y. (2011). Cultivable microbial communities in raw cow milk and potential transfers from stables of sixteen French farms. Int. J. Food Microbiol. 146, 253–262. doi: 10.1016/j.ijfoodmicro.2011.02.033, PMID: 21429612

[ref106] Verdier-Metz I. Gagne G. Bornes S. Monsallier F. Veisseire P. Delbès-Paus C.. (2012). Cow teat skin, a potential source of diverse microbial populations for cheese production. Appl. Environ. Microbiol. 78, 326–333. doi: 10.1128/AEM.06229-11, PMID: 22081572 PMC3255753

[ref107] Wang Y. Nan X. Zhao Y. Jiang L. Wang M. Wang H.. (2021). Rumen microbiome structure and metabolites activity in dairy cows with clinical and subclinical mastitis. J. Anim. Sci. Biotechnol. 12, 36. doi: 10.1186/s40104-020-00543-1, PMID: 33557959 PMC7869221

[ref108] Wang Y. Nan X. Zhao Y. Wang H. Wang M. Jiang L.. (2020). Coupling 16S rDNA sequencing and untargeted mass spectrometry for milk microbial composition and metabolites from dairy cows with clinical and subclinical mastitis. J. Agric. Food Chem. 68, 8496–8508. doi: 10.1021/acs.jafc.0c03738, PMID: 32633125

[ref109] Ward L. M. Hemp J. Shih P. M. McGlynn S. E. Fischer W. W. (2018). Evolution of Phototrophy in the Chloroflexi phylum driven by horizontal gene transfer. Front. Microbiol. 9:260. doi: 10.3389/fmicb.2018.00260, PMID: 29515543 PMC5826079

[ref110] Watts J. L. Lowery D. E. Teel J. F. Rossbach S. (2000). Identification of Corynebacterium bovis and other coryneforms isolated from bovine mammary glands. J. Dairy Sci. 83, 2373–2379. doi: 10.3168/jds.S0022-0302(00)75126-5, PMID: 11049082

[ref111] Welch C. B. Lourenco J. M. Davis D. B. Krause T. R. Carmichael M. N. Rothrock M. J.. (2020). The impact of feed efficiency selection on the ruminal, cecal, and fecal microbiomes of Angus steers from a commercial feedlot. J. Anim. Sci. 98. doi: 10.1093/jas/skaa230, PMID: 32687166 PMC7392532

[ref112] Welch C. B. Lourenco J. M. Krause T. R. Seidel D. S. Fluharty F. L. Pringle T. D.. (2021). Evaluation of the fecal bacterial communities of Angus steers with divergent feed efficiencies Across the lifespan From weaning to slaughter. Front. Vet. Sci. 8:597405. doi: 10.3389/fvets.2021.597405, PMID: 34268344 PMC8275654

[ref113] Wesley I. V. Wells S. J. Harmon K. M. Green A. Schroeder-Tucker L. Glover M.. (2000). Fecal shedding of campylobacter and *Arcobacter* spp. in dairy cattle. Appl. Environ. Microbiol. 66, 1994–2000. doi: 10.1128/AEM.66.5.1994-2000.2000, PMID: 10788372 PMC101445

[ref114] Widyastuti Y. Febrisiantosa A. Tidona F. (2021). Health-promoting properties of lactobacilli in fermented dairy products. Front. Microbiol. 12:673890. doi: 10.3389/fmicb.2021.673890, PMID: 34093496 PMC8175972

[ref115] Williams J. E. Carrothers J. M. Lackey K. A. Beatty N. F. Brooker S. L. Peterson H. K.. (2019). Strong multivariate relations exist among milk, oral, and fecal microbiomes in mother-infant dyads during the first six months postpartum. J. Nutr. 149, 902–914. doi: 10.1093/jn/nxy299, PMID: 31063198 PMC6543206

[ref116] Xu Q. Qiao Q. Gao Y. Hou J. Hu M. Du Y.. (2021). Gut microbiota and their role in health and metabolic disease of dairy cows. Front. Nutr. 8:701511. doi: 10.3389/fnut.2021.701511, PMID: 34422882 PMC8371392

[ref117] Xue M. Sun H. Wu X. Guan L. L. Liu J. (2018). Assessment of rumen microbiota from a large dairy cattle cohort reveals the pan and Core bacteriomes contributing to varied phenotypes. Appl. Environ. Microbiol. 84:e00970-18. doi: 10.1128/AEM.00970-18, PMID: 30054362 PMC6146982

[ref118] Xue M.-Y. Xie Y.-Y. Zhong Y. Ma X.-J. Sun H.-Z. Liu J.-X. (2022). Integrated meta-omics reveals new ruminal microbial features associated with feed efficiency in dairy cattle. Microbiome 10, 32. doi: 10.1186/s40168-022-01228-9, PMID: 35172905 PMC8849036

[ref119] Yu D. Banting G. Neumann N. F. (2021). A review of the taxonomy, genetics, and biology of the genus *Escherichia* and the type species *Escherichia coli*. Can. J. Microbiol. 67, 553–571. doi: 10.1139/cjm-2020-0508, PMID: 33789061

[ref120] Yu J. Ren Y. Xi X. Huang W. Zhang H. (2017). A novel lactobacilli-based teat disinfectant for improving bacterial communities in the milks of cow teats with subclinical mastitis. Front. Microbiol. 8:1782. doi: 10.3389/fmicb.2017.01782, PMID: 29018412 PMC5622921

[ref121] Zhang T. Mu Y. Zhang D. Lin X. Wang Z. Hou Q.. (2019). Determination of microbiological characteristics in the digestive tract of different ruminant species. Microbiol. Open 8:e00769. doi: 10.1002/mbo3.769, PMID: 30585444 PMC6562135

[ref122] Zhang G. Wang Y. Luo H. Qiu W. Zhang H. Hu L.. (2019). The association between inflammaging and age-related changes in the ruminal and fecal microbiota among lactating Holstein cows. Front. Microbiol. 10:1803. doi: 10.3389/fmicb.2019.01803, PMID: 31447814 PMC6696898

[ref124] Zhao L. Li X. Atwill E. R. Aly S. S. Williams D. R. Su Z. (2022). Dynamic changes in fecal bacterial microbiota of dairy cattle across the production line. BMC Microbiol. 22:132. doi: 10.1186/s12866-022-02549-3, PMID: 35568809 PMC9107139

[ref125] Zhong Y. Xue M. Liu J. (2018). Composition of rumen bacterial community in dairy cows with different levels of somatic cell counts. Front. Microbiol. 9:3217. doi: 10.3389/fmicb.2018.03217, PMID: 30619238 PMC6312127

