# Supplementary material for: Characterization of rumen, fecal, and milk microbiota in lactating dairy cows
Source: Front Microbiol. 2022 Sep 26;13:984119. doi: 10.3389/fmicb.2022.984119 (PMC9549371; doi:10.3389/fmicb.2022.984119)
Supplement: Supplementary file 1 [file Table_1.DOCX]

| **UGA Hi SS #1** | | | | | | |
| --- | --- | --- | --- | --- | --- | --- |
| **Ingredient Name** | **AF lb** | **DM %** | **DM lb** |  | **Nutrient Analysis** | **(DM%)** |
| Sorghum Sudan Sil 12.21 | 54.07 | 34 | 18.38 |  | Dry matter (DM), % | 56.73 |
| ^1^12.16.21 UGA GW SS Mix 1 | 22.6 | 88.43 | 19.99 |  | Net energy of lactation, Mcal/lb | 0.77 |
| Hominy Feed | 10 | 90.5 | 9.05 |  | Crude protein, % of DM | 16.5 |
| Cottonseed, Whole | 4 | 92 | 3.68 |  | Neutral detergent fiber, % of DM | 37.1 |
| Bermuda hay | 1 | 90 | 0.9 |  | Acid detergent fiber, % of DM | 22.37 |
| Total | 91.67 |  | 52 |  | Non-fiber carbohydrate, % of DM | 32.42 |

**Supplemental Table 1.** Composition of total mixed ration used to feed the Holstein cattle.

^1^Composition of 12.16.21 UGA GW SS Mix 1 is displayed in Supplemental table 2.

| **UGA GW SS Mix 1** | | | | | | | |
| --- | --- | --- | --- | --- | --- | --- | --- |
| **Ingredient Name** | **AF lb** | **DM %** | **DM lb** | **% of AF** |  | **Nutrient Analysis** | **(DM%)** |
| Corn, Ground Shelled | 9.99 | 87 | 8.71 | 44.25 |  | Dry matter (DM), % | 88.43 |
| Soybean Meal 48% | 7.49 | 89 | 6.67 | 33.16 |  | Net energy of lactation, Mcal/lb | 0.9 |
| Distillers Grains | 2 | 87.5 | 1.75 | 8.85 |  | Crude protein, % of DM | 26.1 |
| Molasses, Liquid | 0.8 | 75 | 0.6 | 3.54 |  | Neutral detergent fiber, % of DM | 11.56 |
| Calcium Carbonate | 0.74 | 99 | 0.73 | 3.28 |  | Acid detergent fiber, % of DM | 5.44 |
| Sodium Bicarb | 0.5 | 99 | 0.5 | 2.21 |  | Non-fiber carbohydrate, % of DM | 46.71 |
| Nurisol | 0.29 | 98 | 0.28 | 1.28 |  | Fat, % of DM | 5.05 |
| Palmit 80 | 0.2 | 99.5 | 0.2 | 0.88 |  | Calcium, % of DM | 1.73 |
| Salt | 0.17 | 99 | 0.17 | 0.75 |  | Phosphorus, % of DM | 0.44 |
| Diamond V XPC | 0.12 | 91 | 0.11 | 0.53 |  | Lysine, % of DM | 1.2 |
| Urea | 0.1 | 99 | 0.1 | 0.44 |  | Methionine, % of DM | 0.36 |
| Magnesium Oxide 58% | 0.08 | 95 | 0.08 | 0.35 |  | Potassium, % of DM | 1.01 |
| Godfrey Warehouse Trace | 0.04 | 98 | 0.04 | 0.19 |  | Magnesium, % | 0.43 |
| Godfrey ADE | 0.04 | 95 | 0.03 | 0.15 |  | Sulfur, % | 0.32 |
| Availa-4 | 0.02 | 98 | 0.02 | 0.07 |  | Added Salt, % | 0.84 |
| Selenium Yeast 600 | 0.02 | 97 | 0.01 | 0.07 |  |  |  |
| Total | 22.6 |  | 19.99 | 100 |  |  |  |

**Supplemental Table 2.** Composition of the concentrate used to feed the Holstein cattle.

|  | **Milk Range^1^** | | | |
| --- | --- | --- | --- | --- |
| **Item** | **Low** | **Medium** | **High** | ***P*-value** |
| OBS_features | 524.67 | 578.50 | 550.80 | 1 |
| Faith's PD^2^ | 54.26 | 46.20 | 52.97 | 0.423 |
| Shannon Index | 7.093 | 7.250 | 7.259 | 1 |
| Evenness | 0.8032 | 0.7960 | 0.8029 | 1 |

**Supplemental Table 3.** Alpha-diversity indices calculated for the milk samples of Holstein dairy cattle with different milk yield ranges at time of collection: low, medium, and high.

^1^Low = Yield ≤ 65 lbs/day. Medium = 65 lbs < Yield < 90 lbs. High = Yield ≥ 90 lbs/day. ^2^Faith’s Phylogenetic Diversity. None of the means within each row were significantly different (P ≥ 0.05) according to Bonferroni’s multiple comparisons.

|  | **Milk Range^1^** | | | |
| --- | --- | --- | --- | --- |
| **Item** | **Low** | **Medium** | **High** | ***P*-value** |
| OBS_features | 1500.93 | 1397.38 | 1359.38 | 0.972 |
| Faith's PD^2^ | 64.36 | 61.56 | 59.50 | 0.979 |
| Shannon Index | 9.576 | 9.431 | 9.275 | 1 |
| Evenness | 0.9086 | 0.9037 | 0.8930 | 1 |

**Supplemental Table 4.** Alpha-diversity indices calculated for the rumen samples of Holstein dairy cattle with different milk yield ranges at time of collection: low, medium, and high.

^1^Low = Yield ≤ 65 lbs/day. Medium = 65 lbs < Yield < 90 lbs. High = Yield ≥ 90 lbs/day. ^2^Faith’s Phylogenetic Diversity. None of the means within each row were significantly different (P ≥ 0.05) according to Bonferroni’s multiple comparisons.

**Supplemental Table 5.** Alpha-diversity indices calculated for the fecal samples of Holstein dairy cattle with different milk yield ranges at time of collection: low, medium, and high.

|  | **Milk Range^1^** | | | |
| --- | --- | --- | --- | --- |
| **Item** | **Low** | **Medium** | **High** | ***P*-value** |
| OBS_features | 1005.43 | 1024 | 921.63 | 0.208 |
| Faith's PD^2^ | 40.35 | 40.91 | 37.99 | 0.316 |
| Shannon Index | 8.844 | 8.851 | 8.704 | 0.838 |
| Evenness | 0.8876 | 0.8856 | 0.8862 | 1 |

^1^Low = Yield ≤ 65 lbs/day. Medium = 65 lbs < Yield < 90 lbs. High = Yield ≥ 90 lbs/day. ^2^Faith’s Phylogenetic Diversity. None of the means within each row were significantly different (P ≥ 0.05) according to Bonferroni’s multiple comparisons.
