# Supplementary material for: Characterization of rumen, fecal, and milk microbiota in lactating dairy cows
Source: Front Microbiol. 2022 Sep 26;13:984119. doi: 10.3389/fmicb.2022.984119 (PMC9549371; doi:10.3389/fmicb.2022.984119)
Supplement: Supplementary file 2 [file Table_2.pdf]

Supplemental Table 1. Composition of total mixed ration used to feed the Holstein cattle.

Ingredient Name	AF[1] lb	DM[2] %	DM lb	Nutrient Analysis	(DM%)
Sorghum Sudan Sil[3] 12.21	54.07	34	18.38	Dry matter (DM), %	56.73
12.16.21 UGA GW SS Mix 1[4]	22.6	88.43	19.99	Net energy of lactation, Mcal/lb	0.77
Hominy Feed	10	90.5	9.05	Crude protein, % of DM	16.5
Cottonseed, Whole	4	92	3.68	Neutral detergent fiber, % of DM	37.1
Bermuda hay	1	90	0.9	Acid detergent fiber, % of DM	22.37
Total	91.67		52	Non-fiber carbohydrate, % of DM	32.42

[1]AF = As fed
[2]DM = Dry matter
[3]Sorghum Sudan Sil = Sorghum Sudan Silage
[4]Composition of 12.16.21 UGA GW SS Mix 1 is displayed in Supplemental table 2.

Supplemental Table 2. Composition of UGA GW SS Mix 1.

Ingredient Name	AF[1] lb	DM[2] %	DM lb	% of AF	Nutrient Analysis	(DM%)
Corn, Ground Shelled	9.99	87	8.71	44.25	Dry matter (DM), %	88.43
Soybean Meal 48%	7.49	89	6.67	33.16	Net energy of lactation, Mcal/lb	0.9
Distillers Grains	2	87.5	1.75	8.85	Crude protein, % of DM	26.1
Molasses, Liquid	0.8	75	0.6	3.54	Neutral detergent fiber, % of DM	11.56
Calcium Carbonate	0.74	99	0.73	3.28	Acid detergent fiber, % of DM	5.44
Sodium Bicarb	0.5	99	0.5	2.21	Non-fiber carbohydrate, % of DM	46.71
Nurisol	0.29	98	0.28	1.28	Fat, % of DM	5.05
Palmit 80	0.2	99.5	0.2	0.88	Calcium, % of DM	1.73
Salt	0.17	99	0.17	0.75	Phosphorus, % of DM	0.44
Diamond V XPC	0.12	91	0.11	0.53	Lysine, % of DM	1.2
Urea	0.1	99	0.1	0.44	Methionine, % of DM	0.36
Magnesium Oxide 58%	0.08	95	0.08	0.35	Potassium, % of DM	1.01
Godfrey Warehouse Trace	0.04	98	0.04	0.19	Magnesium, %	0.43
Godfrey ADE	0.04	95	0.03	0.15	Sulfur, %	0.32
Availa-4	0.02	98	0.02	0.07	Added Salt, %	0.84
Selenium Yeast 600	0.02	97	0.01	0.07		
Total	22.6		19.99	100		

[1]AF = As fed

[2]DM = Dry matter

Supplemental Table 3. Beta-diversity pairwise permanovas

Group 1	Group 2	Permutations	pseudo-F	p-value	q-value
Fecal	Milk	999	41.16603061	0.001	0.001
Fecal	Rumen	999	137.5434827	0.001	0.001
Milk	Rumen	999	52.59921935	0.001	0.001

Supplemental Table 4. Alpha-diversity indices calculated for the milk samples of Holstein dairy cattle with different milk yield ranges at time of collection: low, medium, and high.

	Milk Range[1]			
**Item**	**Low**	**Medium**	**High**	***P*-value**
OBS_features	524.67	578.50	550.80	1
Faith's PD[2]	54.26	46.20	52.97	0.423
Shannon Index	7.093	7.250	7.259	1
Evenness	0.8032	0.7960	0.8029	1

[1]Low: Yield ≤ 65 lbs/day. Medium: 65 lbs < Yield < 90 lbs. High: Yield ≥ 90 lbs/day. [2]Observed features. [3]Faith’s Phylogenetic Diversity. None of the means within each row were significantly different (P ≥ 0.05) according to Bonferroni’s multiple comparisons.

Supplemental Table 5. Alpha-diversity indices calculated for the rumen samples of Holstein dairy cattle with different milk yield ranges at time of collection: low, medium, and high.

	Milk Range[1]			
**Item**	**Low**	**Medium**	**High**	***P*-value**
OBS_features	1500.93	1397.38	1359.38	0.972
Faith's PD[2]	64.36	61.56	59.50	0.979
Shannon Index	9.576	9.431	9.275	1
Evenness	0.9086	0.9037	0.8930	1

[1]Low: Yield ≤ 65 lbs/day. Medium: 65 lbs < Yield < 90 lbs. High: Yield ≥ 90 lbs/day. [2]Observed features. [3]Faith’s Phylogenetic Diversity. None of the means within each row were significantly different (P ≥ 0.05) according to Bonferroni’s multiple comparisons.

Supplemental Table 6. Alpha-diversity indices calculated for the fecal samples of Holstein dairy cattle with different milk yield ranges at time of collection: low, medium, and high.

	**Milk Range[1]**			
**Item**	**Low**	**Medium**	**High**	***P*-value**
OBS_features	1005.43	1024	921.63	0.208
Faith's PD[2]	40.35	40.91	37.99	0.316
Shannon Index	8.844	8.851	8.704	0.838
Evenness	0.8876	0.8856	0.8862	1

[1]Low: Yield ≤ 65 lbs/day. Medium: 65 lbs < Yield < 90 lbs. High: Yield ≥ 90 lbs/day. [2]Observed features. [3]Faith’s Phylogenetic Diversity. None of the means within each row were significantly different (P ≥ 0.05) according to Bonferroni’s multiple comparisons.
